# Integrative GWAS and RNA-Seq Analysis Prioritizes a Major Chromosome 4H Locus and Candidate Genes for Barley Leaf Length

**DOI:** 10.3390/plants15213334

**Published:** 2026-10-28

**Authors:** Panpan Li, Dan Zhang, Zhiguo Xiang, Xianlin Zhao, Zhidan Zuo, Hongshan Yang, Dongyang Liu, Yongying Zhao

**Affiliations:** Wheat Institute, Henan Academy of Agricultural Sciences (HAAS), Zhengzhou 450002, China; li_pp1203@163.com (P.L.);

**Keywords:** barley, leaf-length variation, GWAS, RNA-seq, candidate gene

## Abstract

Leaf length is an important component of barley plant architecture, but its genetic basis remains incompletely understood. Here, we integrated genome-wide association study (GWAS), RNA sequencing (RNA-seq), and sequence variation analyses to investigate the genetic and transcriptional basis of barley leaf-length variation. A panel of 198 barley accessions was evaluated across two growing seasons, and contrasting long- and short-leaf accessions were subjected to RNA-seq. GWAS identified a major association signal on chromosome 4H, with a core interval of approximately 941.73 kb defined by local linkage disequilibrium analysis. RNA-seq identified 3507 and 3878 differentially expressed genes (DEGs) in flag leaves and second leaves below the flag leaf, respectively, including 2648 shared DEGs. Functional enrichment and gene set enrichment analyses indicated differences in developmental, signaling, metabolic, and temperature-responsive processes. Integration of GWAS and RNA-seq data prioritized three candidate genes, *LOC123447426*, *LOC123449930*, and *LOC123450878,* with different levels of supporting evidence. *LOC123447426* and *LOC123450878* were located within the core association interval, whereas *LOC123449930* was located in the extended candidate-screening region, approximately 2.2 Mb from the lead SNP. *LOC123447426* showed the strongest integrated support, with evidence from GWAS and differential expression in both leaf positions. Sequence variation, and qRT-PCR analyses provided further support for these candidates. Overall, this study prioritizes a major chromosome 4H region and three candidate genes associated with barley leaf-length variation, providing a basis for further investigation of their potential roles in leaf development. However, the current evidence does not establish causal relationships between these genes and leaf-length variation, and their biological functions remain to be experimentally verified.

## 1. Introduction

Barley (*Hordeum vulgare* L.) is one of the major cereal crops worldwide. It is widely used for animal feed, malting and brewing, and food production [1,2]. Barley has a diploid genome, extensive genetic diversity, and well-characterized genetic resources, making it an important model for genetic and genomic studies within the Triticeae [3]. Advances in high-density genotyping, genome-wide association studies (GWASs), transcriptome sequencing, and genomic resource development have facilitated the investigation of complex agronomic and developmental traits in barley [4,5,6].

Leaf architecture affects light interception, photosynthetic efficiency, canopy structure, and biomass accumulation. Barley exhibits substantial genetic variation in leaf morphology, including leaf length, width, angle, and thickness [7]. Previous quantitative trait locus (QTL) studies have identified genomic regions associated with flag leaf morphology, chlorophyll content, and the length and area of different leaves [8,9]. Studies of leaf thickness have also identified major loci and potential candidate genes [10,11], highlighting the complex genetic basis of barley leaf architecture.

Leaf length is an important component of leaf size and canopy architecture. It is determined by developmental processes such as cell proliferation and expansion, as well as the timing and rate of leaf elongation [7]. Several genes have been associated with barley leaf development. *PHOTOPERIOD-H1 (Ppd-H1)* is associated with natural variation in leaf size, potentially through differences in leaf growth duration and cell number [12]. *NARROW LEAFED DWARF1 (NLD1)* encodes a WUSCHEL-RELATED HOMEOBOX 3 (WOX3) protein involved in lateral organ development, whereas the INDETERMINATE DOMAIN protein BROAD LEAF1 regulates leaf width by restricting lateral proliferation [13,14]. *HvWOX3A* has also been associated with plant height and leaf size [15]. More recently, a multiparent population study identified 24 consensus QTLs for flag leaf length and width and highlighted the contribution of epidermal cell number to flag leaf length variation [16]. Despite these advances, the genetic basis of the natural variation in barley leaf length remains incompletely understood, and the transcriptional processes underlying this variation require further investigation.

GWAS is an effective approach for investigating complex quantitative traits by exploiting historical recombination and natural genetic variation in diverse germplasm. In barley, GWAS has been applied to agronomic, developmental, stress-response, and grain-quality traits, with high-density SNP datasets and diverse germplasm improving its resolution [17,18,19,20,21]. However, GWAS generally identifies associated genomic regions rather than causal genes, making complementary molecular evidence necessary for candidate-gene prioritization. RNA-seq provides such evidence by identifying differentially expressed genes (DEGs) and characterizing transcriptional responses associated with contrasting phenotypes. In barley, transcriptomic analyses have been used to investigate drought and heat responses, nitrogen metabolism, cadmium stress, and flag leaf senescence [22,23,24,25]. Integrating GWAS and transcriptomic data can therefore combine positional and expression evidence to narrow candidate-gene regions, an approach previously applied to barley stress tolerance, yield-related traits, and grain quality [26,27]. Sequence variation analysis provides additional evidence by identifying natural SNPs and InDels that may contribute to phenotypic differences, which is particularly relevant given the extensive allelic diversity of barley germplasm [28,29].

In this study, we aimed to investigate the genetic basis of natural variation in barley leaf length by integrating phenotypic evaluation, GWAS, RNA-seq, and sequence variation analyses. We hypothesized that natural variation in barley leaf length is associated with genetic loci and differences in gene expression, and that integrating GWAS and transcriptomic evidence would help prioritize candidate genes associated with this trait.

## 2. Results

### 2.1. Phenotypic Variation in Leaf Length in the Barley Population

A panel of 198 barley accessions was evaluated for leaf length (LL) and leaf width (LW) in 2021 and 2022. Substantial phenotypic variation in LL was observed across the association panel in both years (Figure 1A,B). The mean LL was 18.35 cm in 2021, with a range of 10.00–26.80 cm and a coefficient of variation (CV) of 17.77%. In 2022, the mean LL was 14.85 cm, with a range of 7.00–26.67 cm and a CV of 22.30% (Table 1). The frequency distributions of LL were approximately normal in both years. The skewness values were −0.20 in 2021 and 0.35 in 2022. The corresponding kurtosis values were −0.16 and 0.31, respectively (Table 1).

Leaf width (LW) also showed continuous phenotypic variation across the association panel. The mean LW was 1.54 cm in 2021 and 1.22 cm in 2022. The corresponding CVs were 24.02% and 30.54%, respectively (Figure 1C,D; Table 1). These results indicated that both LL and LW varied across the 198 barley accessions, with LL showing a broad distribution in both years.

To further characterize contrasting leaf-length phenotypes, two accessions, D64 and D360, were selected for comparison. D64 had a significantly longer leaf than D360, with LL values of 19.29 ± 0.90 cm and 13.44 ± 0.70 cm, respectively (*p* < 0.01; Table 2; Figure 1E). The two accessions also differed significantly in plant height, number of filled grains per panicle, and 1000-grain weight. D360 showed higher values for these three traits than D64. No significant difference in seed-setting rate was observed between the two accessions. These results confirmed that D64 and D360 had clearly contrasting leaf-length phenotypes. They were therefore selected as contrasting materials for subsequent transcriptome analysis.

### 2.2. Genome-Wide Association and Linkage Disequilibrium Analyses Identify a Major Leaf-Length Locus in Barley

Genome-wide association analysis was performed to investigate the genetic basis of natural variation in leaf length (LL) across the 198 barley accessions. Significant marker–trait associations were detected on multiple chromosomes, with the strongest association signal located on chromosome 4H (Figure 2A,B). The lead SNP was located at Chr4H: 596,652,626. The quantile–quantile (Q–Q) plot showed a generally good fit between the observed and expected distributions, with a clear deviation in the upper tail (Figure 2B). No obvious systematic inflation was observed.

A regional association analysis was then performed around the major signal on chromosome 4H. The strongest association signal was located within the 596.157–597.099 Mb interval, corresponding to approximately 941.73 kb (Figure 2C). The lead SNP at Chr4H: 596,652,626 showed the strongest association with LL. Several nearby SNPs also showed elevated association signals, indicating a concentrated association region around the lead SNP. The Manhattan plot was evaluated using a significance threshold of −log_10_(*P*) = 6, and the corresponding SNP-level *p*-values and significance thresholds are provided in Supplementary Table S1. Linkage disequilibrium (LD) analysis showed a distinct LD structure in this region, with relatively strong LD among neighboring markers (Figure 2C). Together, the regional association and LD patterns supported the presence of a major LL-associated locus within this chromosome 4H region.

To identify genes that might contribute to the observed association, the genomic region was further extended to approximately 596–599 Mb for preliminary candidate screening. A total of 30 annotated genes were identified within this extended candidate-screening region (Table 3). These genes included genes encoding a pentatricopeptide repeat-containing protein, a predicted protein, cell number regulator 2, auxin-induced protein 5NG4, subtilisin-like protease SBT1.2, calmodulin-like protein CML16, KNUCKLES, O-methyltransferase ZRP4, L-allo-threonine aldolase, and other predicted or functionally annotated proteins. Several genes were located within or close to the core association interval. For example, *LOC123450878* and *LOC123447426* were located within the major association region, while *LOC123449902* was located approximately 9.2 kb downstream of the lead SNP.

The extended region was used to avoid excluding genes located near the core association interval during candidate screening. It should be noted that the approximately 596–599 Mb region represents an extended candidate-screening region, rather than the genome-wide significant association interval. The core association interval was defined as 596.157–597.099 Mb based on the regional association signal. These results identified a major LL-associated locus on chromosome 4H and provided a genomic region for subsequent integration with transcriptome data to prioritize candidate genes.

### 2.3. Differential Expression and Expression Pattern Analysis of Contrasting Leaf-Length Genotypes

To further characterize the transcriptional differences between the contrasting leaf-length accessions, RNA-seq was performed using the flag leaf (FL) and the second leaf below the flag leaf (SL) of D64 and D360, with three biological replicates for each accession–leaf position combination. The number of clean reads per sample ranged from 37.68 to 48.10 million, with total mapping rates ranging from 96.11% to 96.90% and Q30 values ranging from 95.80% to 96.25% (Table A1). Pearson correlation analysis showed high consistency among biological replicates, with correlation coefficients ranging from 0.92 to 1.00 (Figure A1). PCA showed that PC1 and PC2 explained 95.8% and 3.1% of the total variance, respectively, accounting for 98.9% cumulatively. The samples formed distinct clusters according to accession and leaf position, while biological replicates within each group clustered closely together (Figure A2). Differentially expressed genes (DEGs) were identified by comparing D64 with D360 in each leaf position. In the FL comparison, 3507 DEGs were identified between D64_FL and D360_FL. Among these genes, 1579 were upregulated and 1928 were downregulated in D64_FL relative to D360_FL (Figure 3A). In the SL comparison, 3878 DEGs were identified between D64_SL and D360_SL. Of these genes, 1830 were upregulated and 2048 were downregulated in D64_SL relative to D360_SL (Figure 3B).

The identified DEGs were distributed across all seven barley chromosomes in both FL and SL comparisons (Figure 3C). The DEGs were therefore not restricted to the chromosome 4H region identified by GWAS. Both comparisons showed genome-wide distributions of differentially expressed genes.

Hierarchical clustering was performed to examine the expression patterns of the DEGs across the samples. The D64 and D360 samples were clearly separated in both FL and SL datasets, while biological replicates showed similar expression patterns (Figure 4A). The DEGs were further classified into nine expression-pattern clusters, designated G-C1 to G-C9 (Figure 4B). Several clusters showed distinct differences between D64 and D360. The genes in G-C1 to G-C3 showed higher expression levels in D360, whereas the genes in G-C4 to G-C6 showed higher expression levels in D64. The remaining clusters showed other expression patterns across the samples.

A total of 2648 DEGs were shared between the FL and SL comparisons (Figure 3A,B). The remaining DEGs were detected only in the FL or SL comparison. Thus, the two leaf positions shared a substantial number of DEGs, while each leaf position also contained a set of specific DEGs. The shared DEGs represented the common transcriptional differences between D64 and D360 across the two leaf positions.

Overall, transcriptome analysis identified 3507 and 3878 DEGs in the FL and SL comparisons, respectively. These DEGs were distributed across all seven barley chromosomes and showed distinct expression patterns between D64 and D360. The 2648 shared DEGs were subsequently used for functional enrichment analysis.

### 2.4. Functional Enrichment Analysis of Differentially Expressed Genes

To further characterize the DEGs identified between D64 and D360, Gene Ontology (GO) and Kyoto Encyclopedia of Genes and Genomes (KEGG) enrichment analyses were performed for the FL and SL comparisons. In the FL comparison, the enriched GO terms included protein phosphorylation, phosphorylation, response to biotic stimulus, response to external stimulus, protein kinase activity, nucleotide binding, and membrane-related terms (Figure 5A). In the SL comparison, enriched GO terms included protein kinase activity, phosphotransferase activity, nucleotide binding, phosphorylation, response to biotic stimulus, response to external stimulus, and plasma membrane-related terms (Figure 5B). Several GO categories were shared between the two leaf positions, including phosphorylation-related terms, nucleotide binding, and responses to biotic or external stimuli.

KEGG enrichment analysis also identified several significantly enriched pathways in both leaf positions (Figure 5C,D). In the FL comparison, the enriched pathways included starch and sucrose metabolism, glycerophospholipid metabolism, glutathione metabolism, and nitrogen metabolism. In the SL comparison, the enriched pathways included starch and sucrose metabolism, glycerophospholipid metabolism, fatty acid degradation, and nitrogen metabolism. Starch and sucrose metabolism, glycerophospholipid metabolism, and nitrogen metabolism were enriched in both FL and SL.

Gene set enrichment analysis (GSEA) was further performed to examine the distribution of predefined gene sets in the ranked DEG lists. In the SL comparison, the GO gene set “response to temperature stimulus” showed significant negative enrichment, with a normalized enrichment score (NES) of −2.07 and an adjusted *p* value < 0.001 (Figure 6). The genes belonging to this gene set were mainly distributed toward the D360_SL end of the ranked list. The same gene set showed an NES of −1.62 in the FL comparison, with a nominal *p* value < 0.01 but an adjusted *p* value > 0.5 (Figure A3). Thus, the enrichment of this gene set was significant after multiple-testing correction only in the SL comparison.

GSEA was also performed for the KEGG Ribosome gene set. In the FL comparison, the NES was −0.51, with an adjusted *p* value > 0.5. In the SL comparison, the NES was 1.28, with a nominal *p* value < 0.05 but an adjusted *p* value > 0.5 (Figure A3). Therefore, the Ribosome gene set did not show significant enrichment after multiple-testing correction in either leaf position.

Overall, GO and KEGG analyses identified several enriched functional categories and metabolic pathways in both FL and SL. GSEA further identified significant enrichment of the “response to temperature stimulus” gene set in SL after multiple-testing correction. These enrichment results provided the functional categories for subsequent analysis of transcription factors, alternative splicing, and putative protein–protein interaction networks.

### 2.5. Transcription Factor Analysis Reveals Putative Regulatory Features Associated with Leaf-Length Variation

To further characterize the molecular features of the DEGs, transcription factor (TF), alternative splicing (AS), and protein–protein interaction (PPI) analyses were performed for the FL and SL comparisons. Several TF families were identified among the DEGs in both leaf positions, including bHLH, C2H2, NAC, B3, MYB, AP2/ERF, WRKY, and MADS-box families (Figure 7A). The composition of differentially expressed TF families varied between FL and SL. In the FL comparison, the major differentially expressed TF families included bHLH, C2H2, NAC, and B3. In the SL comparison, MYB-related, bHLH, C2H2, B3, and HD-ZIP families were among the major differentially expressed TF families. Both upregulated and downregulated TFs were detected in the two leaf positions (Figure 7B,C).

Alternative splicing analysis was then performed to examine transcript isoform variation between D64 and D360. Five major types of AS events were detected, including alternative 3′ splice site (A3SS), alternative 5′ splice site (A5SS), skipped exon (SE), retained intron (RI), and mutually exclusive exon (MXE) events (Figure 8A,B). SE events were the most abundant AS type in both FL and SL, followed by A3SS and A5SS events. Both known and novel AS events were detected in the two leaf positions. The numbers and distributions of AS events differed between D64 and D360 and between FL and SL.

Putative PPI networks were further constructed based on the proteins encoded by the DEGs. The predicted networks showed different interaction patterns between the FL and SL comparisons (Figure 8C,D). Several genes occupied relatively central positions in the predicted networks, with multiple predicted interactions with other proteins. These interactions represent computational predictions and require experimental validation.

Taken together, the DEGs showed differences in TF family composition, AS event distribution, and putative PPI network structure between the contrasting leaf-length accessions. These results provided additional molecular information for the subsequent integration of GWAS and transcriptome data to prioritize candidate genes.

### 2.6. Integration of GWAS and RNA-Seq Identifies Candidate Genes Associated with Barley Leaf Length

To further prioritize candidate genes associated with LL variation, the GWAS results were integrated with the RNA-seq data. The extended candidate-screening region surrounding the major chromosome 4H association locus contained 30 annotated genes (Table 3). These genes were compared with the DEGs identified in the FL and SL transcriptome datasets. A total of 3507 DEGs were identified in the FL comparison and 3878 DEGs were identified in the SL comparison, including 2648 DEGs shared between the two leaf positions.

Three genes within the extended candidate-screening region were identified by integrating the GWAS and RNA-seq results. *LOC123450878* was located in the GWAS region and was differentially expressed between D64 and D360 in FL. *LOC123449930* was located in the GWAS candidate-screening region and was differentially expressed in SL. *LOC123447426* was located in the GWAS region and was differentially expressed in both FL and SL (Figure 9).

Based on the combined evidence, these three genes were prioritized as candidate genes. *LOC123447426* showed the strongest transcriptomic support because it was differentially expressed in both FL and SL. *LOC123449930* showed differential expression in SL, whereas *LOC123450878* showed differential expression in FL. Thus, the three candidate genes were supported by both genetic association and transcriptomic evidence, although the strength and leaf-position specificity of the transcriptomic evidence differed among the three genes. The three prioritized candidate genes were selected for subsequent sequence variation and expression analyses. These analyses were used to further examine the natural variation and expression patterns of *LOC123447426*, *LOC123449930*, and *LOC123450878*.

### 2.7. Natural Sequence Variation and Expression Analysis of Prioritized Candidate Genes

To further characterize the three prioritized candidate genes, sequence variation and expression analyses were performed for *LOC123447426*, *LOC123449930*, and *LOC123450878*. Sixty barley accessions, including thirty long-leaf and thirty short-leaf accessions, were selected for candidate-gene sequencing. Sequence analysis identified multiple natural polymorphisms in all three genes (Figure 10, Figure 11 and Figure 12). Several SNPs were detected across the analyzed region of *LOC123447426*. Multiple SNPs were also identified in *LOC123449930*, including polymorphisms within the coding region. In *LOC123450878*, both SNPs and InDels were detected, including variants near the start codon and within the coding region. These results indicated that the three candidate genes contained natural sequence variation among the selected barley accessions.

The expression patterns of the three candidate genes were further examined by qRT-PCR using FL and SL tissues from D64 and D360. All three genes showed significantly higher expression in D64 than in D360 in both leaf positions (Figure 13). In FL, the relative expression levels of *LOC123447426*, *LOC123449930*, and *LOC123450878* were 0.59, 0.79, and 0.72 in D64, respectively, compared with 0.02, 0.05, and 0.01 in D360. In SL, the corresponding expression levels were 0.61, 0.94, and 0.44 in D64, compared with 0.02, 0.10, and 0.01 in D360. The expression differences between D64 and D360 were significant for all three genes in both FL and SL (Figure 13).

The qRT-PCR results were consistent with the RNA-seq expression patterns of the three candidate genes. Together with their positions within the GWAS candidate region and their differential expression between the contrasting accessions, the sequence variation and expression results provided additional evidence supporting *LOC123447426*, *LOC123449930*, and *LOC123450878* as candidate genes associated with natural variation in barley LL. These findings provide a basis for further investigation of their potential roles in LL variation.

## 3. Discussion

### 3.1. Natural Variation and Genetic Architecture of Barley Leaf Length

Leaf length is an important component of barley plant architecture, affecting light interception, photosynthetic performance, and biomass accumulation. The phenotypic variation among 198 barley accessions across two growing seasons, together with the continuous trait distribution and relatively high coefficients of variation, supports the quantitative nature of leaf length. This is consistent with previous studies highlighting the genetic and environmental influences on barley leaf size [7,9]. Studies of leaf elongation, flag leaf size, peduncle length, and plant height have also demonstrated substantial genetic variation in barley plant architecture [16,30,31]. However, phenotypic variation alone cannot distinguish genetic effects from environmental influences, underscoring the need for integrated genetic and transcriptomic analyses to identify candidate genes associated with leaf length.

GWAS in the present study identified a major association signal on chromosome 4H, with the peak marker located at approximately 596.65 Mb. Local LD analysis defined a core association interval of approximately 941.73 kb. This result provides a genomic region for further investigation of the genetic basis of leaf-length variation. Previous barley studies have identified loci associated with leaf length and other leaf-related traits on several chromosomes, indicating that leaf size is genetically complex [9,32]. Digel et al. (2016) identified *Ppd-H1* as an important locus associated with leaf size and demonstrated that natural variation at this locus affected the duration of leaf growth and leaf cell number [12]. Studies of barley leaf thickness have also identified multiple QTLs and candidate genes associated with leaf morphology and yield-related traits [11]. The 4H locus identified here therefore adds another candidate genomic region to the genetic architecture of barley leaf length.

The phenotypic contrast between D64 and D360 provided an additional basis for transcriptome analysis. The two accessions differed markedly in leaf length and also showed differences in several agronomic traits. These correlated phenotypic differences should be interpreted cautiously because the present experiments do not establish whether the 4H locus or any of the candidate genes have pleiotropic effects on these traits. Nevertheless, the strong leaf-length contrast between D64 and D360 provided a suitable genetic background for identifying transcriptional differences between contrasting materials. However, these differences may also reflect variation in genetic background, developmental status, or other physiological traits, rather than leaf length alone. Together, the GWAS and phenotypic analyses indicate that natural variation in barley leaf length can be investigated by combining population-level genetic mapping with transcriptomic comparison of contrasting materials.

### 3.2. Transcriptomic Differences Reveal Biological Processes Associated with Leaf-Length Variation

RNA-seq analysis revealed extensive transcriptional differences between D64 and D360 in both flag leaves and second leaves. A total of 3507 DEGs were identified between D64_FL and D360_FL, whereas 3878 DEGs were detected between D64_SL and D360_SL. Importantly, 2648 DEGs were shared between the two leaf positions. The large number of shared DEGs suggests that the contrasting leaf phenotypes were accompanied by common transcriptional differences across different leaf positions. At the same time, the presence of leaf-position-specific DEGs indicates that part of the transcriptional response may depend on developmental or physiological context. However, because D64 and D360 also differed in other agronomic traits, these transcriptional differences cannot be attributed specifically to leaf-length variation.

Leaf size is determined by the coordinated regulation of cell proliferation and cell expansion. In grasses, leaf growth involves continuous production and expansion of cells within the basal growth zone. Thus, genetic differences in developmental timing, cell proliferation, or cell expansion can contribute to variation in final leaf size [7,12]. In Arabidopsis and other plant systems, cell-cycle activity, hormone signaling, and cell-expansion processes are closely integrated during organ growth [33,34]. The regulation of leaf development also involves coordinated auxin and cytokinin signaling [35,36]. Auxin can promote rapid cell expansion, whereas cytokinins contribute to cell proliferation and developmental patterning [33,35]. The enrichment of phosphorylation- and protein kinase-related functions observed in the present study is therefore consistent with differences in signaling activity between long- and short-leaf materials. However, these enrichment patterns do not by themselves demonstrate that these signaling processes directly determine barley leaf length.

The enrichment of metabolic functions, including starch and sucrose metabolism and glycerophospholipid-related processes, provides additional information about the physiological differences between D64 and D360. Carbohydrate metabolism is closely associated with the supply and utilization of carbon during plant growth, whereas membrane lipid metabolism is relevant to cellular membrane structure and function. Such changes may accompany differences in leaf development and physiological status. Hormonal pathways may also be involved because auxin, cytokinins, and other phytohormones participate in leaf growth and development [33,35,37]. Auxin-related responses are diverse and can affect cell expansion, cell division, and developmental patterning [38]. Gibberellins have also been implicated in the regulation of leaf development and growth [37]. Therefore, the transcriptomic data support a model in which leaf-length variation is associated with coordinated differences in developmental signaling and cellular metabolism. Further physiological and cellular analyses will be required to determine which of these processes are directly related to the observed leaf-length differences.

### 3.3. Environmental Response and Regulatory Features Provide Additional Molecular Context

The GSEA results provided an additional layer of information beyond conventional DEG enrichment analysis. Among the tested gene sets, the GO term “response to temperature stimulus” showed significant negative enrichment in D64_SL compared with D360_SL, with an NES of −2.07 and an adjusted *p* value < 0.001. The same gene set showed a similar direction of enrichment in FL, but the association did not remain significant after multiple-testing correction. Thus, the most robust GSEA result was detected in SL. This difference between leaf positions suggests that environmental-response-related transcription may vary according to developmental context.

Temperature is an important environmental factor affecting plant development, and environmental signals can interact with the cell-cycle machinery and developmental programs that determine organ growth [34]. Barley leaf growth is also influenced by developmental transitions and environmental conditions, as demonstrated for photoperiod-dependent regulation of leaf size [32]. More broadly, molecular networks involved in leaf development integrate developmental signals with environmental information to coordinate cell proliferation and organ growth [39,40]. Therefore, the temperature-response enrichment observed here may indicate that environmental-response processes are associated with the transcriptomic differences between long- and short-leaf materials. However, the current experiment does not allow us to determine whether temperature-responsive genes directly contribute to natural variation in leaf length. The GSEA result should therefore be considered as molecular context rather than direct evidence of a temperature-mediated leaf-length mechanism.

Transcription factor analysis further revealed differences in several TF families, including bHLH, C2H2, NAC, B3, MYB, AP2/ERF, WRKY, and MADS. Transcription factors coordinate developmental signals and downstream gene-expression programs, and multiple TF families participate in plant organ development and environmental responses. The enrichment of these TF families in the DEG set therefore suggests that transcriptional regulation may contribute to the molecular differences between D64 and D360. Plant transcription factors function within complex regulatory networks, and their effects depend on spatial and temporal context as well as interactions with other regulatory proteins [41]. Nevertheless, TF enrichment alone does not identify direct TF–target relationships. The putative regulatory relationships inferred from the present transcriptomic data should therefore be experimentally validated.

Alternative splicing analysis provided another layer of regulatory information. Multiple types of alternative splicing events were detected in both FL and SL, indicating that transcript isoform diversity accompanies the transcriptional differences between the two materials. Alternative splicing can modulate transcript abundance, protein structure, and developmental responses and is widely recognized as an important component of plant regulatory plasticity [42,43]. Recent work has also emphasized the importance of intron processing and splice-site regulation in determining the biological relevance of alternative transcripts [44]. However, the current analysis does not establish whether any individual splice variant contributes to leaf-length variation. Similarly, the predicted protein–protein interaction networks provide hypotheses concerning functional relationships among DEGs but should not be interpreted as experimentally confirmed interactions. Together, the GSEA, TF, alternative splicing, and PPI analyses provide complementary molecular context for the observed leaf-length variation and identify regulatory processes that warrant further investigation.

### 3.4. Integrative GWAS and Transcriptome Analysis Prioritizes Three Candidate Genes Associated with Leaf-Length Variation

The integration of GWAS and transcriptomic data provided an effective strategy for prioritizing candidate genes associated with natural variation in barley leaf length. GWAS can identify genomic regions associated with quantitative traits, but the relatively large number of genes within an associated interval can make candidate prioritization challenging. Transcriptomic information provides an independent layer of evidence by identifying genes with differential expression between contrasting phenotypes. Nevertheless, such expression differences may also reflect genetic-background, developmental, or physiological differences between the selected accessions and should not be interpreted as direct evidence of leaf-length-specific effects. Integrative GWAS and transcriptome analyses have therefore been used to narrow candidate gene lists for complex traits in barley. For example, combined GWAS and transcriptome analyses have been applied to identify candidate genes associated with waterlogging tolerance and salt-response-related traits in barley [26,45,46]. These studies illustrate the utility of integrating genetic association and transcriptional evidence for candidate-gene prioritization.

In the present study, three genes within the candidate genomic region showed additional support from transcriptomic analysis and were prioritized for further investigation. These genes were designated *LOC123447426*, *LOC123449930*, and *LOC123450878*, respectively. Importantly, the three genes were supported by different combinations of genetic and transcriptomic evidence. *LOC123447426* was detected in the GWAS region and was differentially expressed in both FL and SL, whereas *LOC123449930* and *LOC123450878* were supported by GWAS combined with differential expression in SL and FL, respectively. Thus, *LOC123447426* showed the strongest integrated evidence among the three candidates, while *LOC123449930* and *LOC123450878* provided additional candidates supported by complementary datasets.

The stronger evidence for *LOC123447426* is particularly notable because its differential expression was consistently detected in both leaf positions. Such consistency may indicate that the association of *LOC123447426* with leaf-length variation is not restricted to a single sampled leaf position. However, differential expression alone does not establish a causal relationship between gene activity and leaf length. The predicted KNUCKLES-like annotation of *LOC123447426* provides an additional functional clue. KNUCKLES-related genes have been associated with the regulation of meristem activity and developmental processes in Arabidopsis [47]. KNUCKLES function is related to the maintenance and activity of stem-cell-associated developmental programs [47,48]. Because leaf growth depends on coordinated cell proliferation and cell expansion, the annotation of *LOC123447426* is compatible with a potential role in developmental processes related to leaf growth. Nevertheless, the functional similarity between the barley candidate and its Arabidopsis counterpart remains to be experimentally established. Therefore, *LOC123447426* should currently be considered a high-priority candidate gene associated with leaf-length variation rather than a confirmed regulator of this trait.

*LOC123450878* was supported by the GWAS signal and differential expression in FL. Its annotation as a cell number regulator provides a biologically relevant clue because differences in final organ size can arise from changes in cell proliferation and cell number. Previous studies in barley have shown that genetic variation in leaf size can be associated with differences in leaf growth and cellular characteristics [12,16]. More broadly, cell-cycle activity is closely connected with organ growth and development in plants [34,40]. The relationship between cell number and organ size has also been demonstrated in maize, where CELL NUMBER REGULATOR1 affects plant and organ size [49]. Thus, the predicted function of *LOC123450878* is consistent with a potential association with leaf-length variation. However, this interpretation is based on functional annotation and integrated omics evidence, and direct experimental evidence for the role of *LOC123450878* in barley leaf development is still lacking.

*LOC123449930* was supported by the GWAS signal and differential expression in SL. The gene was annotated as a respiratory burst oxidase-like protein B1. This annotation suggests a possible connection with cellular redox-related processes, which can participate in plant signaling and development. However, the current dataset does not directly demonstrate a relationship between *LOC123449930*, reactive oxygen species, and leaf growth. Therefore, the present evidence supports *LOC123449930* as a candidate gene associated with leaf-length variation, but does not establish its molecular function in this process. Further genetic and biochemical analyses will be necessary to determine whether the observed association reflects a functional contribution of *LOC123449930* or linkage with another causal gene or variant.

Sequence variation and expression analyses provided additional support for the three prioritized genes. Naturally occurring sequence variants were detected in all three genes among the selected barley accessions, while qRT-PCR analysis further showed differential expression between the contrasting materials. The convergence of GWAS, transcriptomic, sequence-variation, and expression evidence strengthens the prioritization of these genes. Similar GWAS-based analyses have been used to identify favorable genetic variants and candidate genes for plant organ traits in rice [50,51,52,53]. Nevertheless, these datasets provide evidence at different biological levels and should not be interpreted as equivalent evidence of causality. In particular, sequence variants detected in candidate genes may be linked to causal variants, and expression differences may result from downstream responses to phenotypic differences. Functional validation is therefore required to determine whether specific variants or alleles directly contribute to the observed leaf-length variation.

The different levels of evidence among the three genes also provide a useful basis for subsequent functional studies. *LOC123447426*, which showed support from GWAS and differential expression in both FL and SL, represents the highest-priority candidate for functional validation. *LOC123449930* and *LOC123450878*, supported by GWAS and differential expression in one of the two leaf positions, represent additional candidates that may contribute to leaf-length variation through potentially different biological processes. This prioritization strategy reduces the candidate space while retaining genes supported by independent evidence. Similar integrative approaches have been useful for candidate gene discovery in barley complex traits [26,45,53,54].

Overall, the combined evidence supports *LOC123447426*, *LOC123449930*, and *LOC123450878* as putative candidate genes associated with natural variation in barley leaf length, with *LOC123447426* receiving the strongest support from the current datasets. These findings provide a focused set of targets for subsequent functional characterization and genetic validation. Future studies involving gene editing, transgenic complementation, expression manipulation, and cellular phenotyping will be important for determining whether these candidate genes may contribute to leaf-length variation and for clarifying their potential biological functions.

### 3.5. Limitations and Future Directions

The present study provides an integrated genetic and transcriptomic framework for investigating natural variation in barley leaf length. The identification of a major association locus on chromosome 4H extends the genomic resources available for barley leaf-related traits. The transcriptomic analysis further showed that leaf-length variation was accompanied by extensive differences in developmental, signaling, metabolic, and environmental-response-related gene expression. The integration of GWAS and RNA-seq data then prioritized three candidate genes, *LOC123447426*, *LOC123449930*, and *LOC123450878*. Sequence variation and qRT-PCR provided additional evidence supporting their potential relevance to leaf-length variation.

Several limitations should nevertheless be considered. First, the GWAS identifies statistical associations rather than causal genes or causal variants. Fine mapping and larger genetic populations will be needed to determine the causal genomic variation within the 4H interval. Second, the transcriptome analysis was based on the contrasting accessions D64 and D360. Although this design is useful for identifying genes associated with strong phenotypic differences, the observed expression patterns should be validated across a broader set of barley accessions and developmental stages. Because D64 and D360 differed in other agronomic traits (Table 2), the observed transcriptional differences may also reflect their genetic backgrounds and developmental or physiological status, and cannot be attributed solely to leaf-length variation. Third, the sequence variation identified in the three candidate genes may include linked or non-causal polymorphisms, and the observed differences in sequence variants do not establish a causal relationship with leaf length. Promoter activity assays, allele-specific expression analysis, and targeted mutagenesis would help distinguish functional variants from linked polymorphisms and further clarify their potential contributions to leaf-length variation.

The regulatory information generated by TF, alternative splicing, and PPI analyses also requires experimental validation. Candidate TF–target relationships could be examined using yeast one-hybrid assays, dual-luciferase reporter assays, or chromatin-based approaches. Putative protein interactions could be tested using yeast two-hybrid, co-immunoprecipitation, or bimolecular fluorescence complementation assays. Most importantly, CRISPR/Cas-mediated gene editing, overexpression, or complementation experiments will be required to determine whether *LOC123447426*, *LOC123449930*, or *LOC123450878* affects leaf length in barley. Cellular phenotyping would further clarify whether the candidate genes are associated with changes in cell number, cell size, or leaf growth duration.

Overall, the present study supports a model in which natural variation in barley leaf length is associated with a major genomic region on chromosome 4H and coordinated differences in gene expression across leaf positions. The combined GWAS, transcriptomic, sequence-variation, and expression evidence highlights *LOC123447426*, *LOC123449930*, and *LOC123450878* as candidate genes. These findings provide a useful foundation for subsequent functional validation and may facilitate the development of molecular markers for genetic analysis and improvement of barley plant architecture.

## 4. Materials and Methods

### 4.1. Plant Materials and Field Experiments

A panel of 198 barley (*Hordeum vulgare* L.) accessions was used to investigate the natural variation and genetic basis of leaf length. The accessions represented diverse barley germplasm and were evaluated under field conditions in two growing seasons, 2021 and 2022, at the Modern Agricultural Research and Development Base, Henan, China (113.704663° E, 35.005322° N). The 198 barley accessions were sown in October 2020 and October 2021, and leaf-length and leaf-width measurements were taken at the heading stage in April 2021 and April 2022, respectively. The field experiments were conducted using a randomized complete block design. For each accession, 10 individual plants within each plot were sampled at the heading stage. Leaf length (LL) and leaf width (LW) were measured at the heading stage using a ruler. LL was measured from the leaf base to the tip, whereas LW was measured at the widest point of the flag leaf. For each sampled plant, the flag leaf of the main spike was measured. The mean of the measurements from the 10 plants was used for subsequent phenotypic analyses. The phenotypic measurements and leaf sampling for transcriptome analysis were both performed at the heading stage to ensure consistency between the phenotypic and transcriptomic data collection time points.

Two accessions, D64 and D360, showing contrasting leaf-length phenotypes were selected for transcriptomic and subsequent molecular analyses. D64 exhibited a relatively long-leaf phenotype, whereas D360 exhibited a relatively short-leaf phenotype. The two accessions were selected to maximize the contrast in leaf length while minimizing potential differences in genetic background as far as possible. Both accessions were grown under the same field experimental conditions. For candidate-gene sequence variation analysis, 60 accessions, comprising the 30 accessions with the highest and the 30 accessions with the lowest mean leaf-length values across the 2021 and 2022 growing seasons, were selected from the same 198-accession GWAS panel. This set included D64 and D360 and represented a subset of the GWAS panel rather than an independent population.

### 4.2. Phenotypic Data Analysis

Descriptive statistics, including the mean, minimum, maximum, coefficient of variation (CV), skewness, and kurtosis, were calculated for leaf length (LL) and leaf width (LW) in the 198 barley accessions evaluated in 2021 and 2022. The phenotypic distributions of LL and LW were visualized using frequency distribution plots. For GWAS, the arithmetic mean of leaf-length measurements from the 2021 and 2022 growing seasons was calculated for each accession and used as the phenotypic input. GWAS was performed using a mixed linear model (MLM) to account for population structure and kinship. Differences in leaf length between D64 and D360 were evaluated using Student’s *t*-test. Differences in other agronomic traits between the two accessions were assessed using the same test. All statistical analyses were performed using Microsoft Excel (Microsoft Corporation, Redmond, WA, USA; https://www.microsoft.com/microsoft-365/excel; accessed 7 May 2026), and a *p* value < 0.05 was considered statistically significant unless otherwise stated.

### 4.3. Genotyping, GWAS, and Linkage Disequilibrium Analysis

Genomic DNA was extracted from the 198 barley accessions using the CTAB method. Genotypic data were generated using double-digest restriction site-associated DNA sequencing (ddRAD-seq) and PE150 sequencing on an Illumina NovaSeq platform (Illumina Inc., San Diego, CA, USA). Raw reads were quality-checked using FastQC v0.20.0 (v2026) and filtered with Trimmomatic v0.39 (v2026). Clean reads were aligned to the barley reference genome Morex v3 using BWA-MEM v0.7.17(v2026), and SNPs were identified using GATK v4.2 (v2026). SNPs were filtered using a minor allele frequency (MAF) ≥ 0.05 and a missing rate ≤ 20%.

Genome-wide association analysis was performed using SNP genotype and leaf-length phenotype data from the barley association panel. The efficient mixed-model association expedited (EMMAX) method was used to identify marker–trait associations, with population structure and kinship included in the model. Manhattan and quantile–quantile (Q–Q) plots were generated to visualize the GWAS results. The significance threshold used in the Manhattan plot was set at −log_10_(*P*) = 6. For additional significance assessment, the Bonferroni-corrected threshold was calculated as *P* = 0.05/N, and the suggestive threshold was calculated as *P* = 1/N, where N is the number of SNPs included in the GWAS. Linkage disequilibrium (LD) analysis was performed around significant association loci using D′ and r^2^. Pairwise r^2^ values between SNPs were calculated using PopLDdecay v3.4 (v2026). A 500 kb window was used as an empirical region for examining local association signals and LD patterns. The LD regions were used to define candidate intervals, and genes within or near these intervals were identified based on the Morex v3 reference genome.

### 4.4. RNA Extraction, RNA Sequencing, and Differential Expression Analysis

Transcriptome profiling was conducted using flag leaves (FL) and second leaves below the flag leaf (SL) collected from D64 and D360 at the heading stage, corresponding to the stage at which leaf-length phenotypes were measured. Three biological replicates were used for each sample group. Following sampling, leaf tissues were rapidly frozen in liquid nitrogen and stored at −80 °C until RNA extraction. Total RNA was isolated with TRIzol reagent (Invitrogen, Carlsbad, CA, USA) according to the manufacturer’s instructions. Libraries were constructed from the extracted RNA and sequenced on an Illumina platform. The filtered clean reads were mapped against the Morex v3 barley reference genome using HISAT2 v2.2.1 (v2026), followed by gene expression quantification with Counts v2.0.1 (v2026). Differentially expressed genes were identified using DESeq2 v1.38.3 (v2026) by comparing D64 and D360 separately in FL and SL. Genes with |log_2_(fold change)| > 1 and an unadjusted *p* value < 0.05 were considered DEGs.

### 4.5. Functional Enrichment Analysis

Gene Ontology (GO) and Kyoto Encyclopedia of Genes and Genomes (KEGG) enrichment analyses were performed to characterize the functional categories and biological pathways associated with the differentially expressed genes (DEGs). Enrichment analyses were conducted using clusterProfiler v4.6.0 (v2026) based on the corresponding barley gene annotation. Multiple-testing correction was performed using the Benjamini–Hochberg method, and an adjusted *p* value < 0.05 was considered statistically significant. Gene set enrichment analysis (GSEA) was performed using ranked gene-expression data to assess the enrichment of predefined GO and KEGG gene sets between D64 and D360. Normalized enrichment scores (NES), nominal *p* values, and adjusted *p* values were calculated for each gene set.

Gene Ontology (GO) and Kyoto Encyclopedia of Genes and Genomes (KEGG) enrichment analyses were performed to characterize the functional categories and biological pathways associated with the differentially expressed genes (DEGs). Enrichment analyses were conducted using clusterProfiler v4.6.0 based on the corresponding barley gene annotations provided by the sequencing service provider (Shanghai Personal Biotech-nology Co., Ltd., Shanghai, China). Enrichment significance was assessed using the hypergeometric test, with *p* < 0.05 considered statistically significant.

Gene set enrichment analysis (GSEA) was performed using clusterProfiler v4.6.0 (v2026) to assess the enrichment of predefined GO and KEGG gene sets based on ranked gene-expression data from comparisons between D64 and D360. Gene sets with |NES| > 1, nominal *p* value (NOM *p* value) < 0.05, and false discovery rate (FDR) *q* value < 0.25 were considered significantly enriched.

### 4.6. Transcription Factor, Novel Transcript, and Alternative Splicing Analyses

For novel transcript analysis, mapped reads were assembled using StringTie v2.2.1 (v2026) based on the barley reference genome. The assembled transcripts were compared with known transcripts, and transcripts with Class Codes “j”, “i”, “u”, and “x” were considered novel transcripts. Functional annotation was performed for the identified novel transcripts. Differential alternative splicing (AS) events were analyzed using rMATS v4.0.1, which identifies five types of AS events: skipped exon (SE), retained intron (RI), mutually exclusive exons (MXE), alternative 5′ splice site (A5SS), and alternative 3′ splice site (A3SS). The analyzed AS event types included skipped exon (SE), retained intron (RI), mutually exclusive exons (MXE), alternative 5′ splice site (A5SS), and alternative 3′ splice site (A3SS). Junction-count results were used for the analysis, and AS events with an FDR < 0.01 and |IncLevelDifference| ≥ 0.05 were considered significant. For transcription factor analysis, protein sequences were compared against the Plant Transcription Factor Database (PlantTFDB) to identify transcription factors and their corresponding families. Differentially expressed transcription factors (DETFs) were identified from the DEGs and classified according to their transcription factor families. The numbers of DETFs in different families were summarized separately for the FL and SL comparisons. Protein–protein interaction (PPI) networks were constructed using the STRING database, and interactions with a combined score > 0.95 were retained for network analysis.

### 4.7. Integration of GWAS and RNA-Seq Data

Genes located within the GWAS-associated genomic regions were compared with the DEGs identified from the RNA-seq analysis. Genes supported by both genetic association and differential expression were retained for further candidate-gene evaluation. The overlap between GWAS-derived genes and RNA-seq-derived DEGs was visualized using a Venn diagram. Candidate genes were further evaluated based on their genomic location, expression patterns, and functional annotations.

### 4.8. Sequence Variation Analysis

To investigate natural sequence variation in the selected candidate genes, 60 accessions (30 long-leaf and 30 short-leaf accessions) were selected from the 198-accession GWAS panel based on the mean leaf-length values across the 2021 and 2022 growing seasons. The 198 accessions were ranked in ascending order of mean leaf length, and the 30 accessions with the lowest mean values and the 30 accessions with the highest mean values were selected as the short-leaf and long-leaf groups, respectively. This set included D64 and D360. Genomic DNA was extracted from young leaf tissues and used for PCR amplification and sequencing of the selected candidate genes. Gene-specific primers were used for amplification, and the primer sequences are listed in Appendix A Table A2. The obtained sequences were compared with the corresponding reference sequences to identify SNPs and InDels within the analyzed regions.

### 4.9. qRT-PCR Validation

To validate the expression patterns identified by RNA-seq, quantitative real-time PCR (qRT-PCR) was performed for the selected candidate genes. Total RNA was extracted from the corresponding leaf tissues using TRIzol reagent. First-strand cDNA was synthesized from total RNA using a reverse transcription kit (Sangon Biotech Co., Ltd., Shanghai, China) according to the manufacturer’s instructions.

Gene-specific primers were used for qRT-PCR, and the primer sequences are listed in Appendix A Table A3. qRT-PCR reactions were performed using TB Green Premix Ex Taq II (Sangon Biotech Co., Ltd., Shanghai, China) according to the manufacturer’s instructions. Actin was used as the internal reference gene. The results of qRT-PCR were calculated according to the formula 2 ^−∆CT^, 2 ^−(CT sample−CT Actin)^, with three biological replicates. The qRT-PCR experiment was independently repeated twice, and one representative experiment was selected for presentation. The means from the three biological replicates were subjected to one-way analysis of variance followed by Duncan’s multiple range test, with *p* ≤ 0.05 considered statistically significant.

## 5. Conclusions

In this study, we integrated phenotypic evaluation, GWAS, RNA-seq, and sequence variation analyses to investigate the genetic basis of barley leaf-length variation. A major association region on chromosome 4H was identified, with a core interval of approximately 941.73 kb defined by local linkage disequilibrium analysis. Transcriptome analysis revealed extensive gene expression differences between contrasting accessions in both flag leaves and second leaves below the flag leaf, providing additional molecular evidence associated with leaf-length variation.

Integration of GWAS and RNA-seq results prioritized three candidate genes, *LOC123447426*, *LOC123449930*, and *LOC123450878*, with different levels of supporting evidence. *LOC123447426* showed the strongest integrated support, being located within the core association interval and differentially expressed in both leaf positions. *LOC123450878* was also located within the core interval and was differentially expressed in flag leaves. In contrast, *LOC123449930* was located in the extended candidate-screening region, approximately 2.2 Mb from the lead SNP, and showed comparatively weaker support. Sequence variation and qRT-PCR analyses provided additional evidence supporting the potential association of these candidates with leaf-length variation. Nevertheless, the current findings do not establish causal relationships between these genes and leaf length or determine their precise molecular functions. Further functional studies, including gene editing, transgenic analysis, and cellular phenotyping, are needed to clarify their biological roles. Overall, this study identifies a major chromosome 4H association region and prioritizes *LOC123447426* and *LOC123450878* within the core interval, together with *LOC123449930* as a more distant candidate, for further investigation of the genetic and molecular basis of barley leaf-length variation.

## Figures and Tables

**Figure 1:**
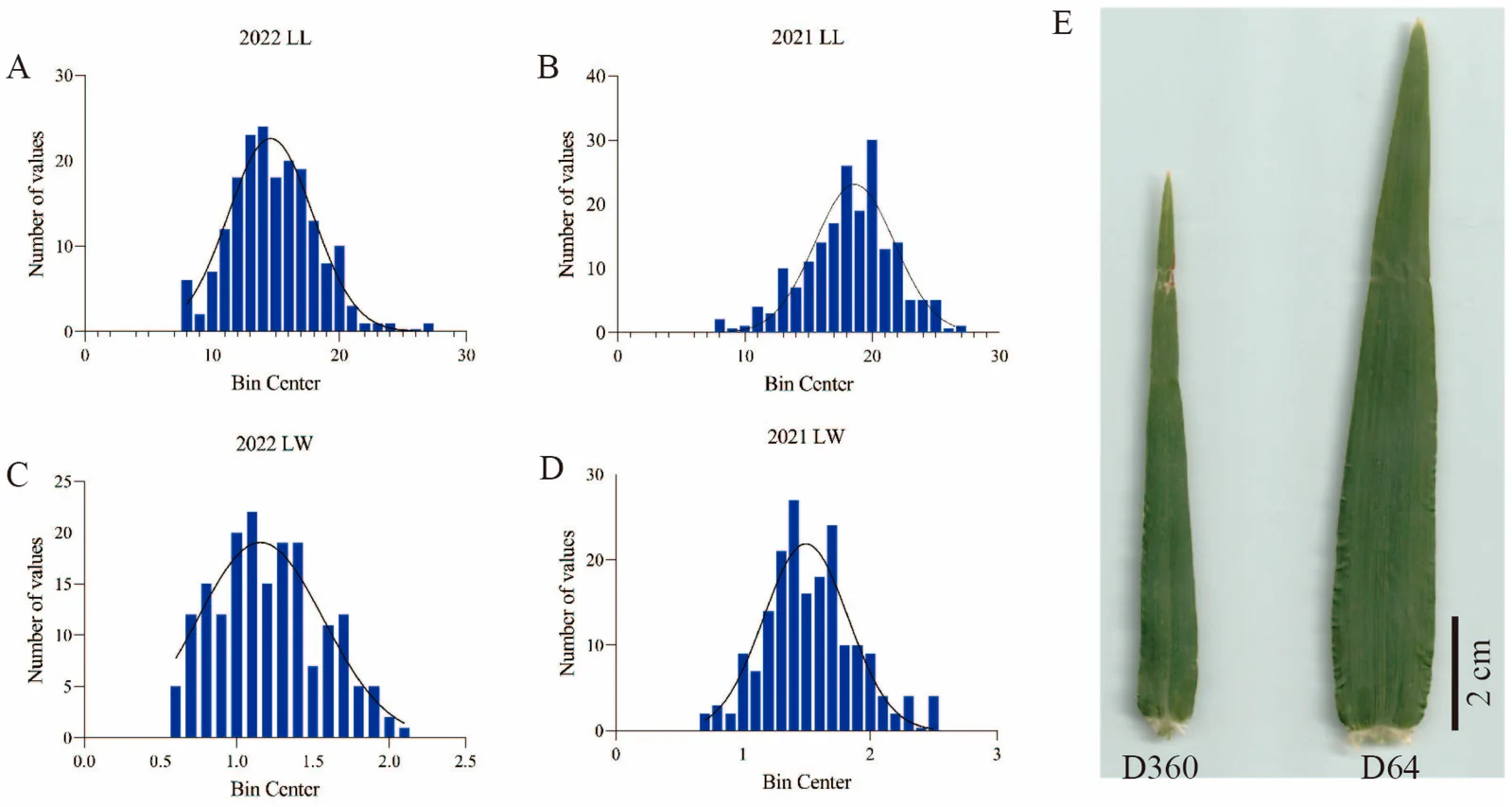
Phenotypic variation and contrasting leaf-length phenotypes in 198 barley accessions. (**A**,**B**) Frequency distributions of leaf length (LL) in 2022 and 2021, respectively. (**C**,**D**) Frequency distributions of leaf width (LW) in 2022 and 2021, respectively. (**E**) Representative leaves of the long-leaf accession D64 and the short-leaf accession D360. Bar = 2 cm.

**Figure 2:**
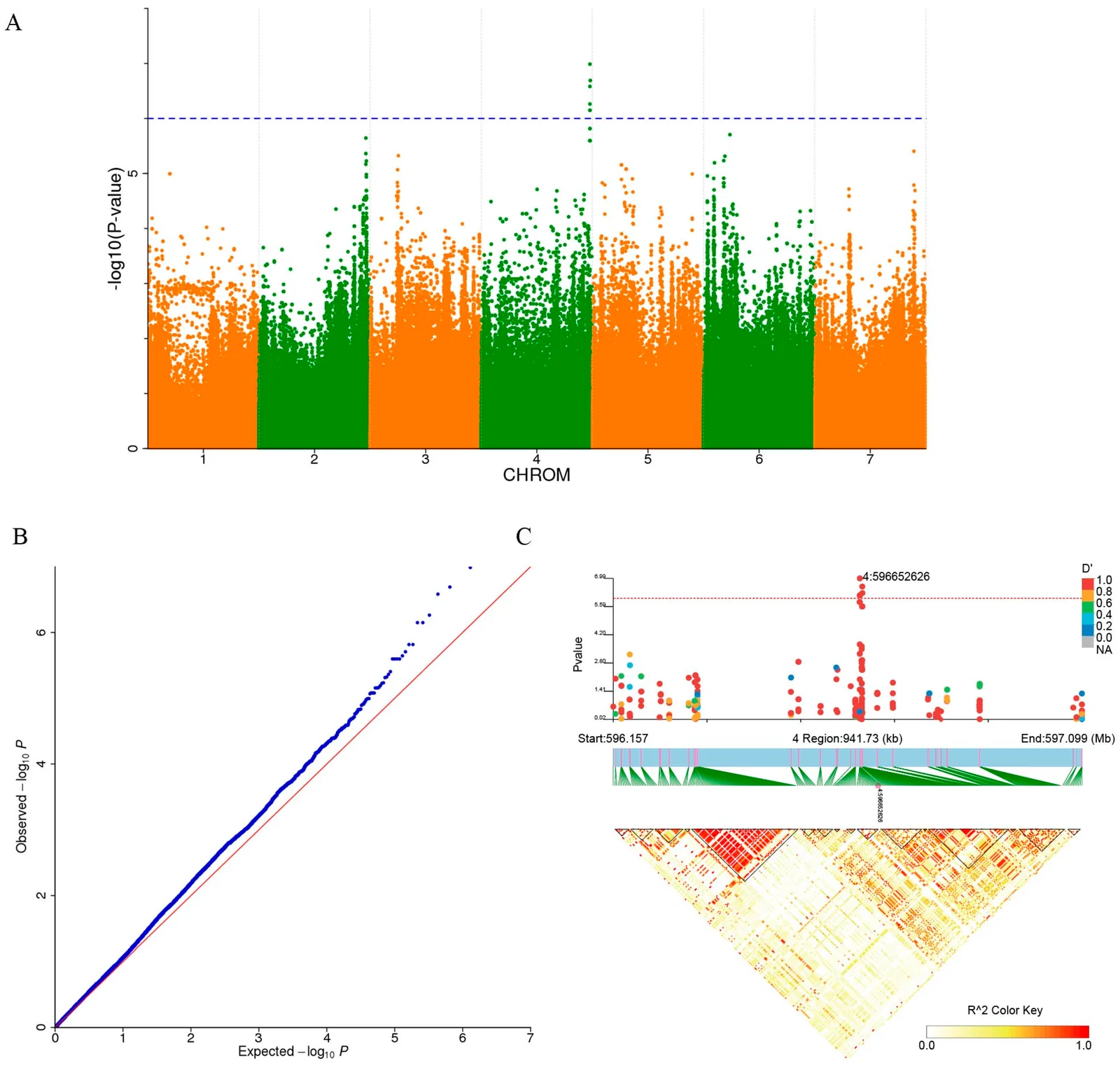
Genome-wide association and linkage disequilibrium analyses of leaf length in barley. (**A**) Manhattan plot showing genome-wide associations with leaf length. Different colors indicate different chromosomes, and the dashed line represents the significance threshold (−log_10_(*P*) = 6). (**B**) Quantile–quantile (Q–Q) plot of the GWAS results. The red line indicates the expected distribution under the null hypothesis. (**C**) Regional association analysis and linkage disequilibrium (LD) pattern surrounding the major leaf-length locus on chromosome 4.

**Figure 3:**
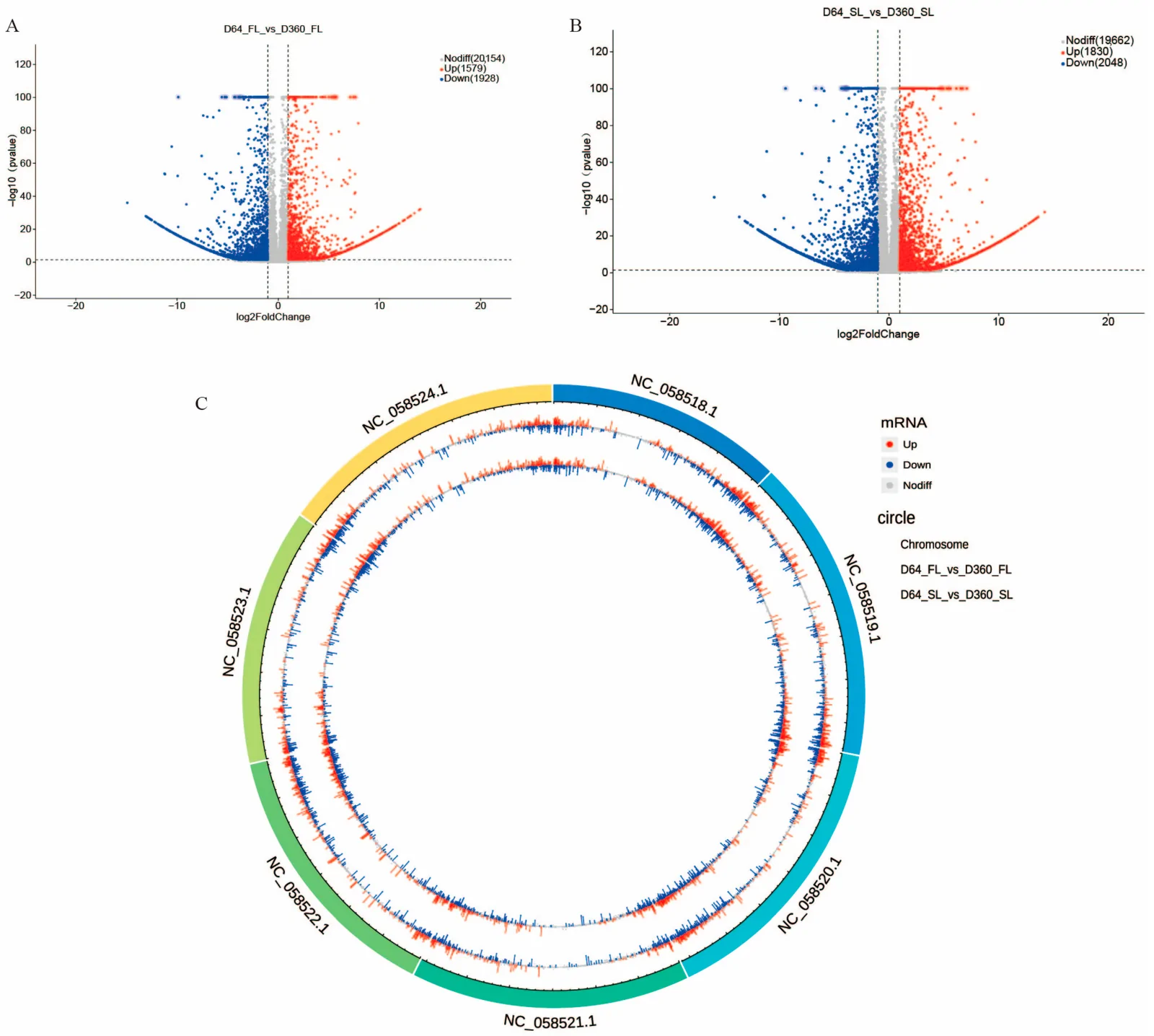
Differentially expressed genes and their genome-wide distribution between contrasting leaf-length barley genotypes. (**A**) Volcano plot of D64_FL vs. D360_FL. (**B**) Volcano plot of D64_SL vs. D360_SL. (**C**) Genome-wide distribution of differentially expressed genes across the seven barley chromosomes. FL, flag leaf; SL, second leaf below the flag leaf. Red, blue, and gray indicate up-regulated, down-regulated, and non-differentially expressed genes, respectively.

**Figure 4:**
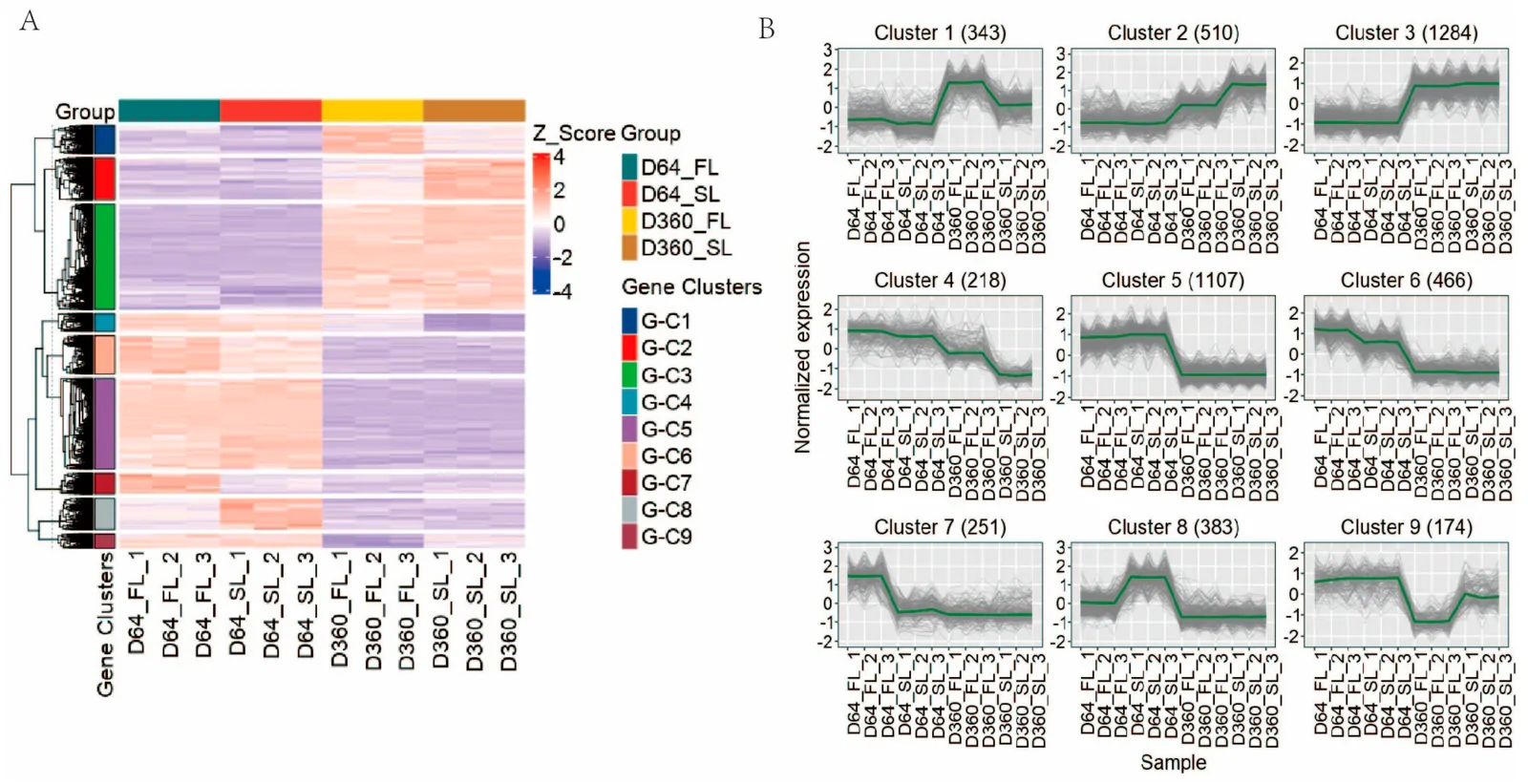
Distinct expression patterns of differentially expressed genes in contrasting leaf-length barley genotypes. (**A**) Clustering analysis of DEGs; (**B**) trend analysis.

**Figure 5:**
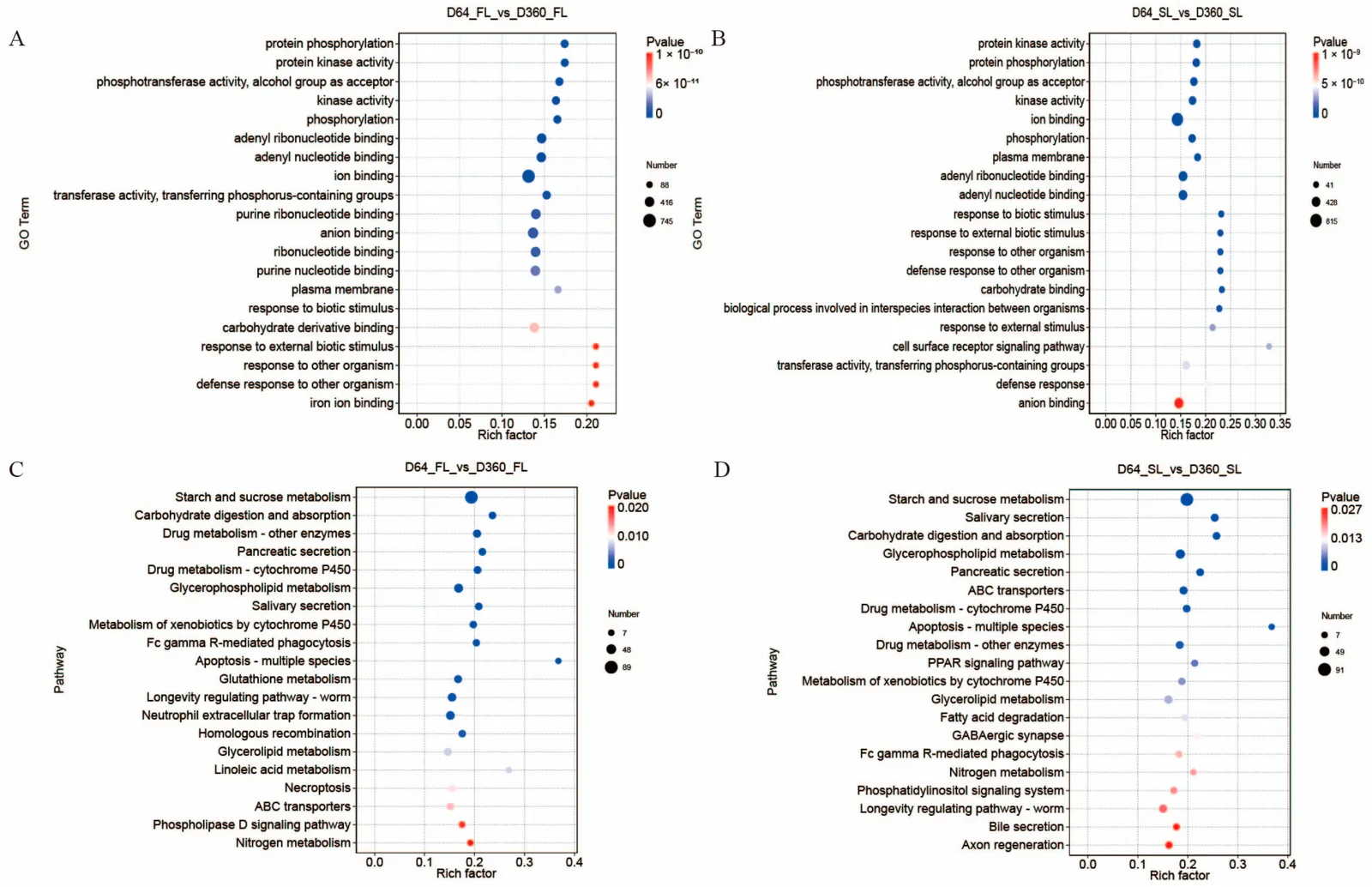
GO and KEGG enrichment analyses of differentially expressed genes between long- and short-leaf barley accessions. (**A**,**B**) GO enrichment analysis of DEGs between D64 and D360 in the flag leaf (FL) and second leaf below the flag leaf (SL). (**C**,**D**) KEGG enrichment analysis of DEGs between D64 and D360 in FL (**C**) and SL (**D**). The *x*-axis represents the rich factor, the size of each dot indicates the number of genes, and the color represents the *p* value.

**Figure 6:**
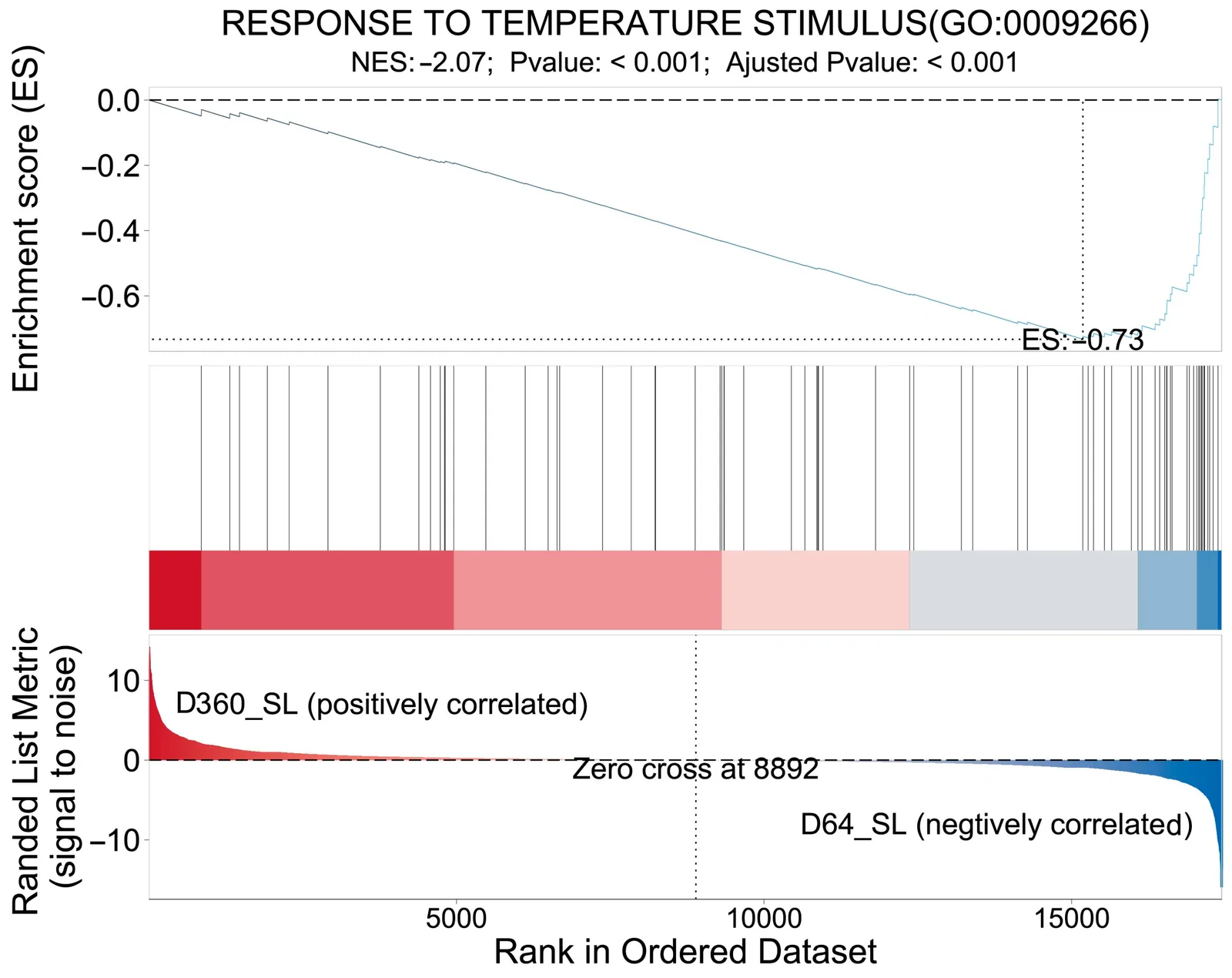
GSEA of the “response to temperature stimulus” gene set in SL. The upper panel shows the running enrichment score (ES), the middle panel indicates the distribution of genes belonging to the gene set within the ranked gene list, and the lower panel shows the ranked list metric for the comparison between D64_SL and D360_SL. The gene set showed a negative enrichment with an NES of −2.07 and an adjusted *p* value < 0.001. The positive and negative sides of the ranked list represent genes positively associated with D360_SL and D64_SL, respectively. Different colors indicate the enrichment score and ranked list metric, as shown in the figure. The dashed line indicates the zero reference level.

**Figure 7:**
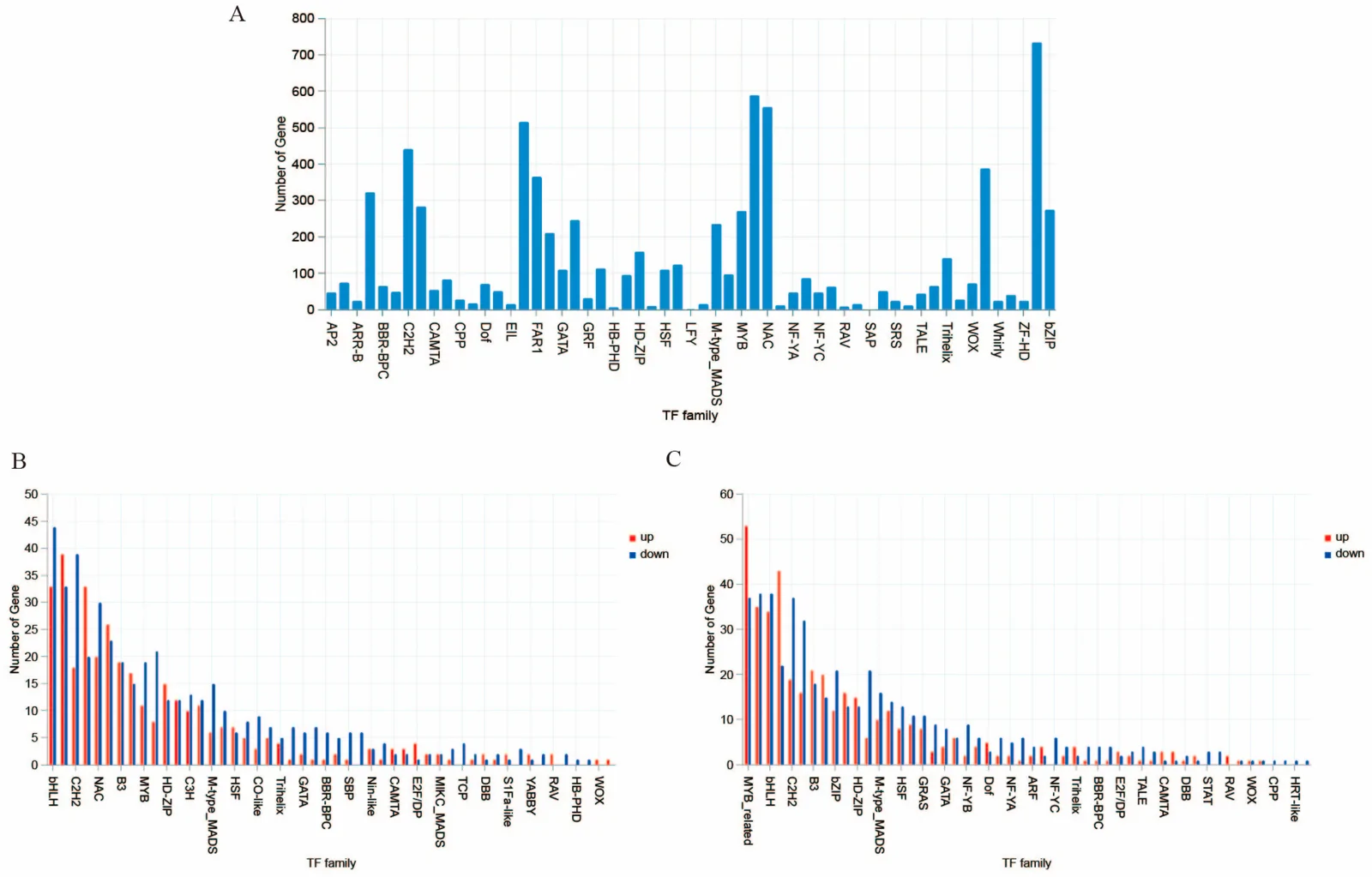
Transcription factor analysis associated with leaf-length variation in barley. (**A**) Distribution of transcription factor families identified in the transcriptome. (**B**,**C**) Distribution of differentially expressed transcription factors in FL (**B**) and SL (**C**).

**Figure 8:**
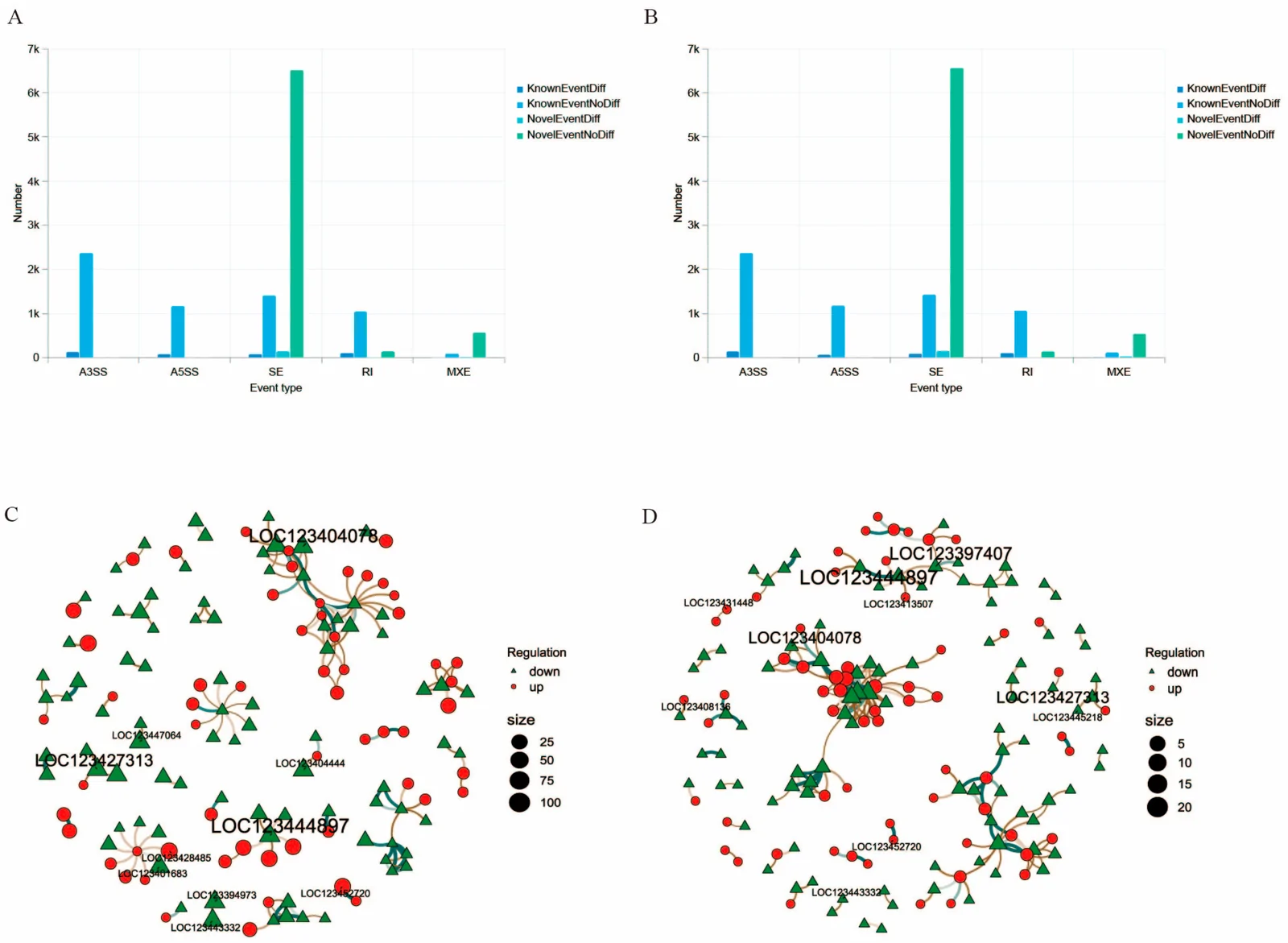
Alternative splicing and putative protein–protein interaction analyses associated with leaf-length variation in barley. (**A**,**B**) Distribution of alternative splicing events in FL (**A**) and SL (**B**). (**C**,**D**) Putative protein–protein interaction networks based on differentially expressed genes in FL (**C**) and SL (**D**).

**Figure 9:**
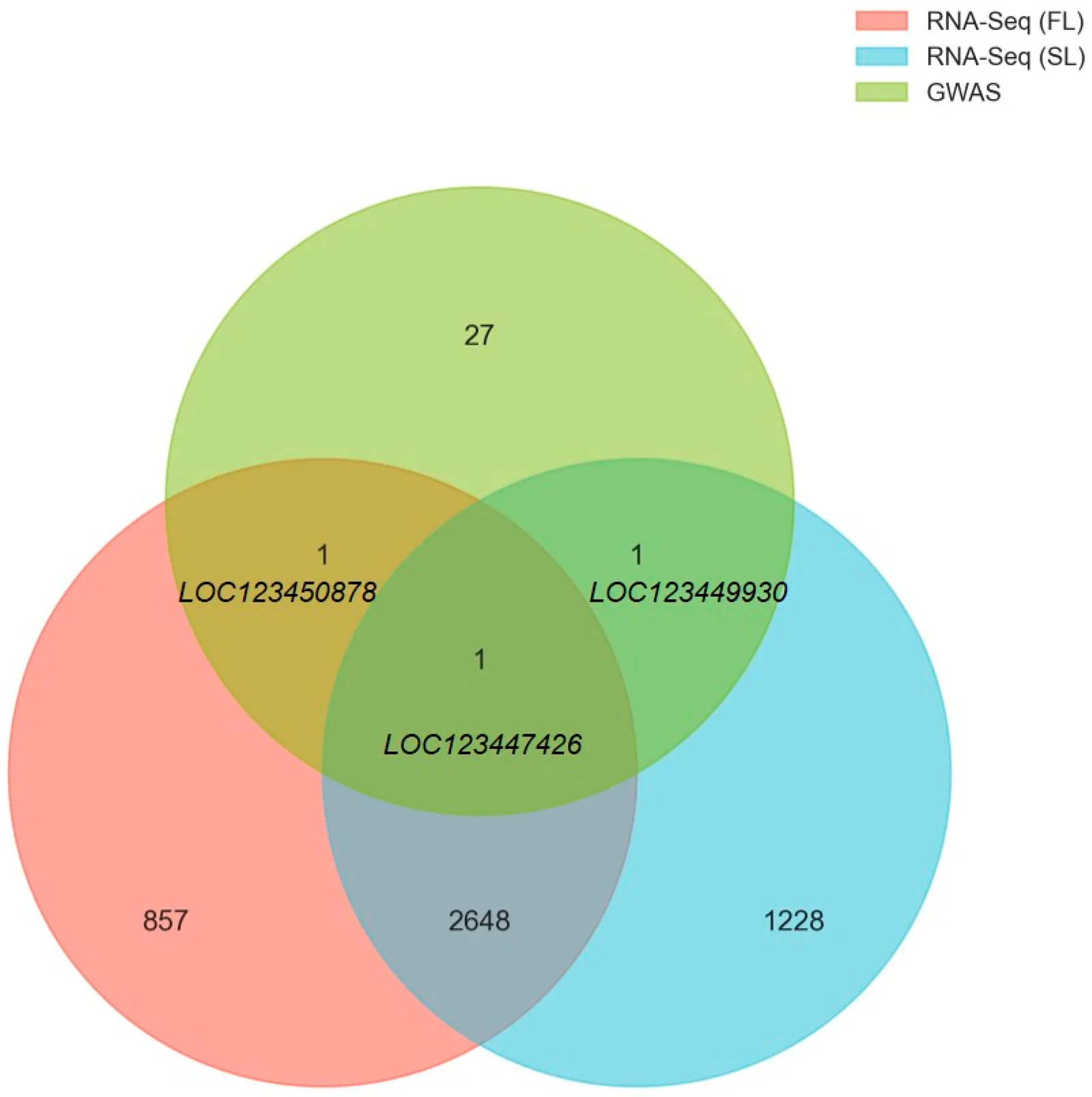
Integration of GWAS and RNA-seq identifies three prioritized candidate genes associated with barley leaf length.

**Figure 10:**
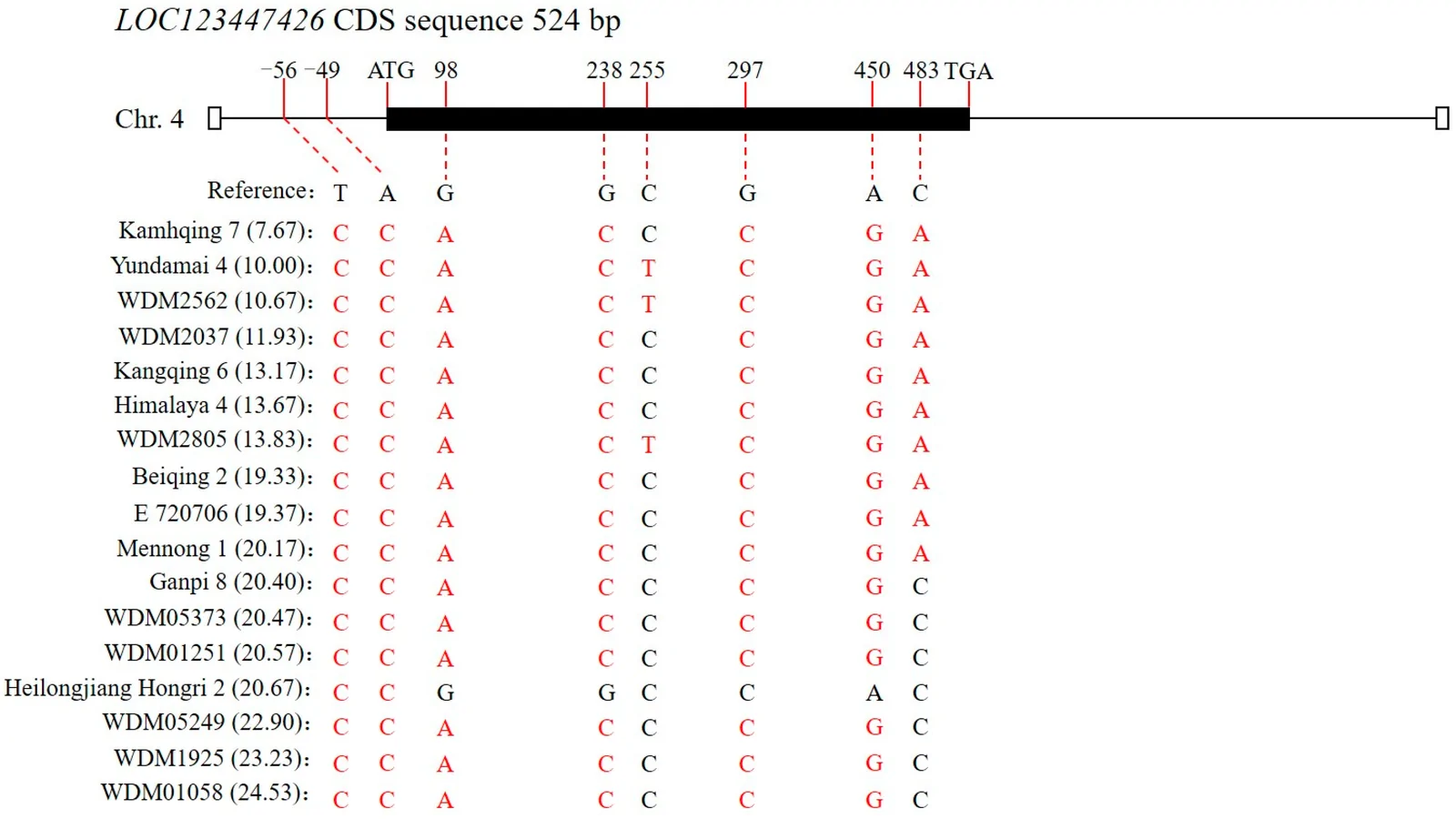
Natural sequence variation analysis of *LOC123447426*.

**Figure 11:**
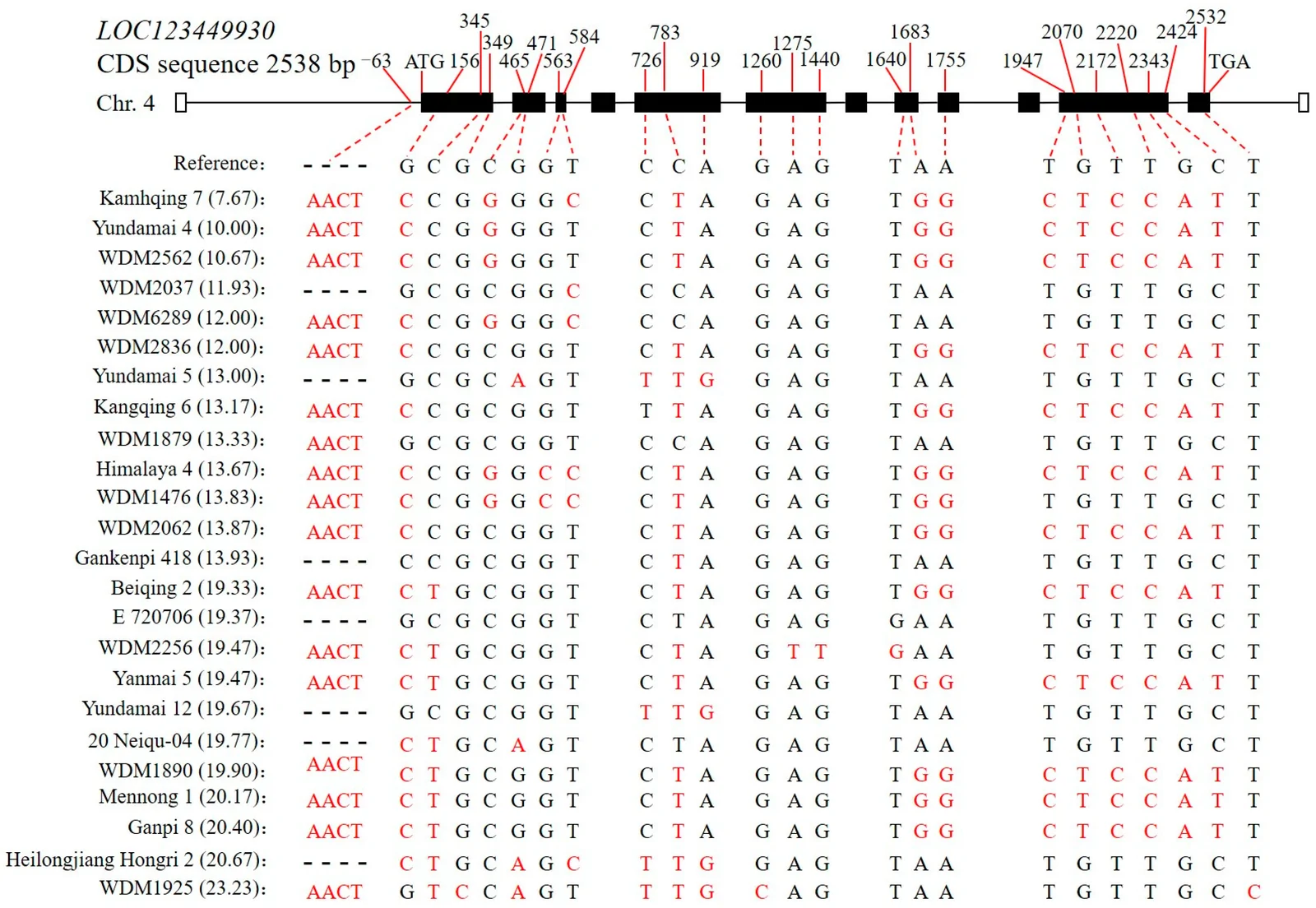
Natural sequence variation analysis of *LOC123449930*.

**Figure 12:**
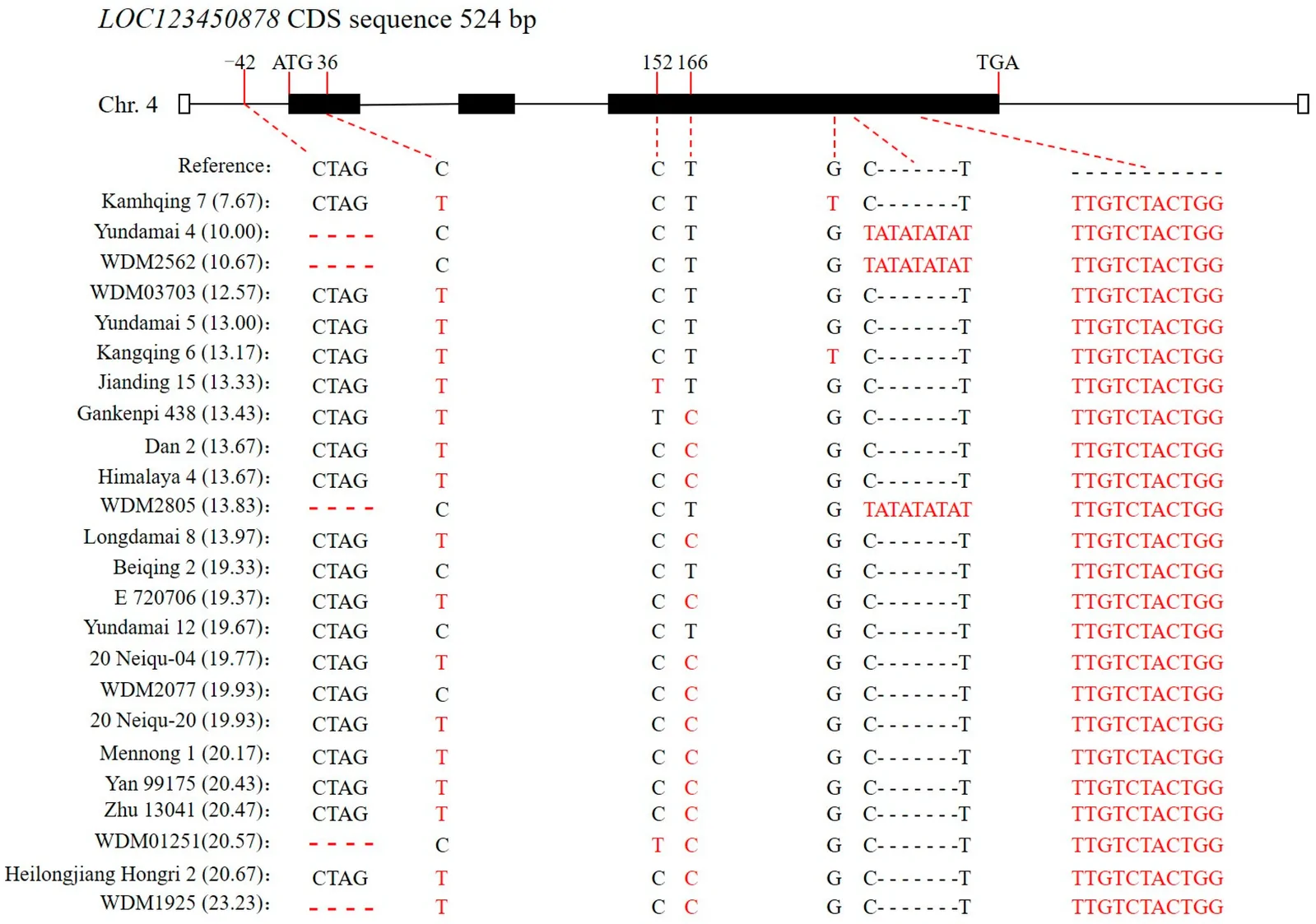
Natural sequence variation analysis of *LOC123450878*.

**Figure 13:**
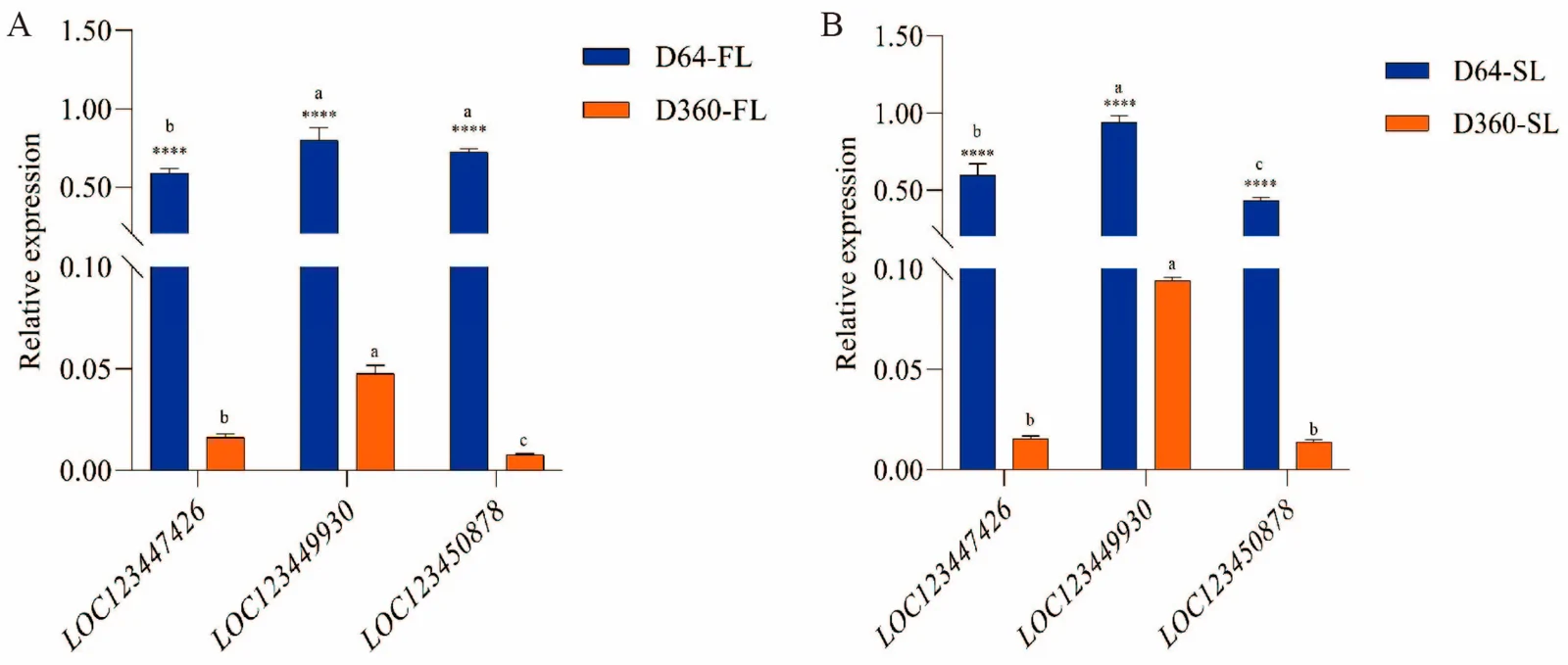
Expression analysis of prioritized candidate genes in long- and short-leaf barley accessions. (**A**) Relative expression levels of *LOC123447426*, *LOC123449930*, and *LOC123450878* in FL. (**B**) Relative expression levels of *LOC123447426, LOC123449930, and LOC123450878* in SL. Different lowercase letters indicate significant differences among groups. Asterisks indicate significant differences between D64 and D360.

**Table 1:** Phenotypic variation and descriptive statistics of leaf length and leaf width in the barley association panel.

Trail	Mean	SD	CV	Range	Skewness	Kurtosis
LL-2021	18.35	3.260	17.765	10.00~26.8	−0.20	−0.16
LL-2022	14.85	3.311	22.303	7.00~26.67	0.35	0.31
LW-2021	1.54	0.369	24.021	0.70~2.83	0.50	0.62
LW-2022	1.22	0.374	30.535	0.50~2.33	0.50	−0.06

**Table 2:** Comparison of agronomic traits between long-leaf and short-leaf barley accessions D64 and D360.

Material	Plant Height (cm)	Leaf Length (cm)	No. Tilles/Plant	No. Filled Grains/Panicle	Seed-Setting (%)	1000-Grain Weight (g)
D64	52.0 ± 1.6	19.29 ± 0.9	8.4 ± 1.1	52.6 ± 10.3	93.67 ± 2.7	49.44 ± 1.6
D360	71.6 ± 2.1 **	13.44 ± 0.7 **	11.6 ± 3.8	25.0 ± 2.8 **	92.04 ± 4.3	68.94 ± 1.6 **

Note: ** indicates a highly significant difference (*p* < 0.01).

**Table 3:** Annotated genes within the extended candidate-screening region surrounding the major chromosome 4H association locus.

Gene-ID	Gene-Start	Gene-End	NR
*LOC123450876*	596271138	596274965	pentatricopeptide repeat-containing protein
*LOC123449899*	596304539	596309218	predicted protein
*LOC123450878*	596331153	596331964	cell number regulator 2
*LOC123450880*	596511400	596517288	Auxin-induced protein 5NG4
*LOC123449902*	596661855	596664539	subtilisin-like protease SBT1.2
*LOC123450881*	596675614	596676336	putative calcium-binding protein CML16
*LOC123447423*	596684874	596688605	predicted protein
*LOC123449903*	596782605	596786291	O-methyltransferase ZRP4
*LOC123449904*	596789224	596792506	L-allo-threonine aldolase
*LOC123450883*	596849146	596851448	protein HOTHEAD-like
*LOC123447425*	596865203	596865940	Zinc finger protein KNUCKLES
*LOC123447426*	596881129	596881693	Zinc finger protein KNUCKLES
*LOC123447428*	597049411	597050166	predicted protein
*LOC123449900*	596284994	596302987	hypothetical protein D1007_44104
*LOC123449901*	596565690	596608332	putative callose synthase
*LOC123449906*	596819854	596821785	uncharacterized protein LOC119292086
*LOC123449907*	596822124	596836519	hydroxymethylglutaryl-CoA synthase-like
*LOC123447459*	598315834	598317544	3′-N-debenzoyl-2′-deoxytaxol N-benzoyltransferase
*LOC123449928*	598467568	598469247	short-chain dehydrogenase TIC 32, chloroplastic-like
*LOC123447461*	598639615	598641162	short-chain dehydrogenase TIC 32, chloroplastic-like
*LOC123447465*	598846342	598848239	predicted protein
*LOC123447466*	598875768	598876243	hypothetical protein D1007_54876
*LOC123447467*	598897567	598900390	CASP-like protein 5B3
*LOC123449931*	598957979	598959877	predicted protein
*LOC123449933*	599076274	599077934	pentatricopeptide repeat-containing protein
*LOC123447471*	599093354	599094606	BTB/POZ and MATH domain-containing protein 2-like
*LOC123450888*	598275614	598302035	E3 ubiquitin-protein ligase WAV3-like
*LOC123449929*	598489705	598491274	short-chain dehydrogenase TIC 32, chloroplastic-like
*LOC123449930*	598865080	598870484	respiratory burst oxidase-like protein B1
*LOC123449932*	598995521	599000354	WD repeat-containing protein 44

**Figure A1:**
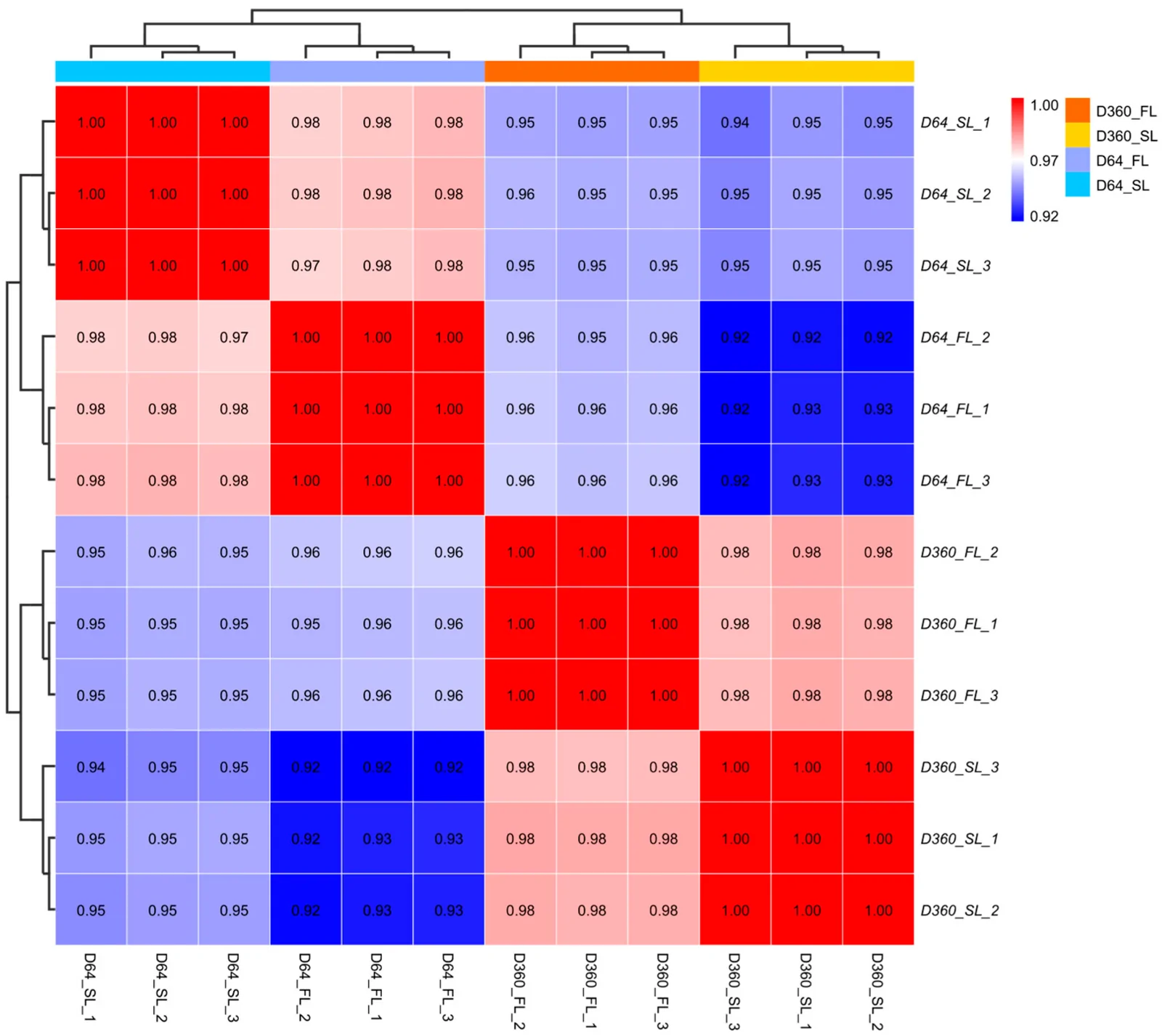
Pearson correlation analysis among RNA-seq biological replicates of D64 and D360 in the flag leaf and second leaf below the flag leaf. The heatmap shows pairwise Pearson correlation coefficients among the 12 samples. FL, flag leaf; SL, second leaf below the flag leaf.

**Figure A2:**
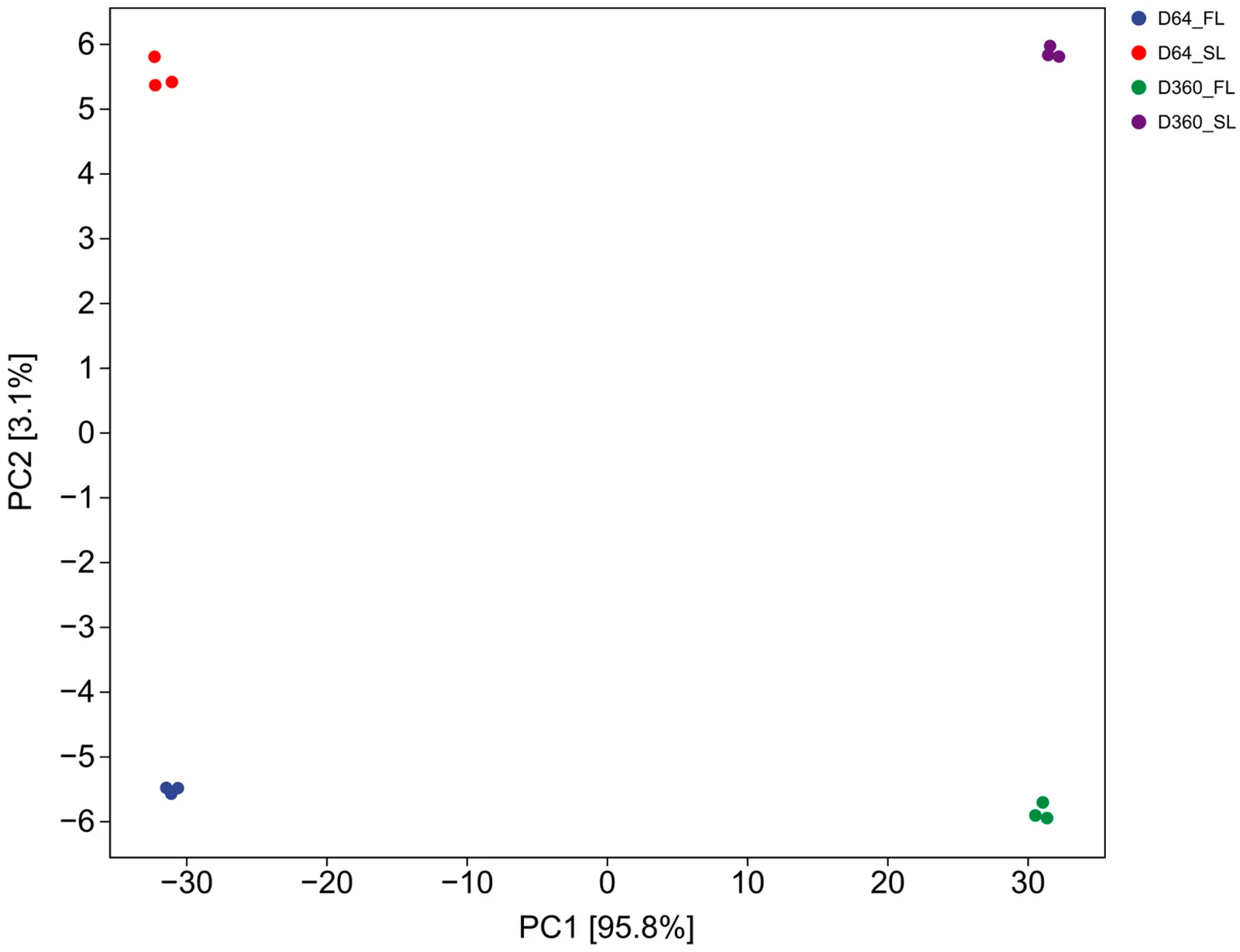
Principal component analysis (PCA) of RNA-seq samples from D64 and D360 in the flag leaf and second leaf below the flag leaf. PC1 and PC2 explain 95.8% and 3.1% of the total variance, respectively. FL, flag leaf; SL, second leaf below the flag leaf.

**Figure A3:**
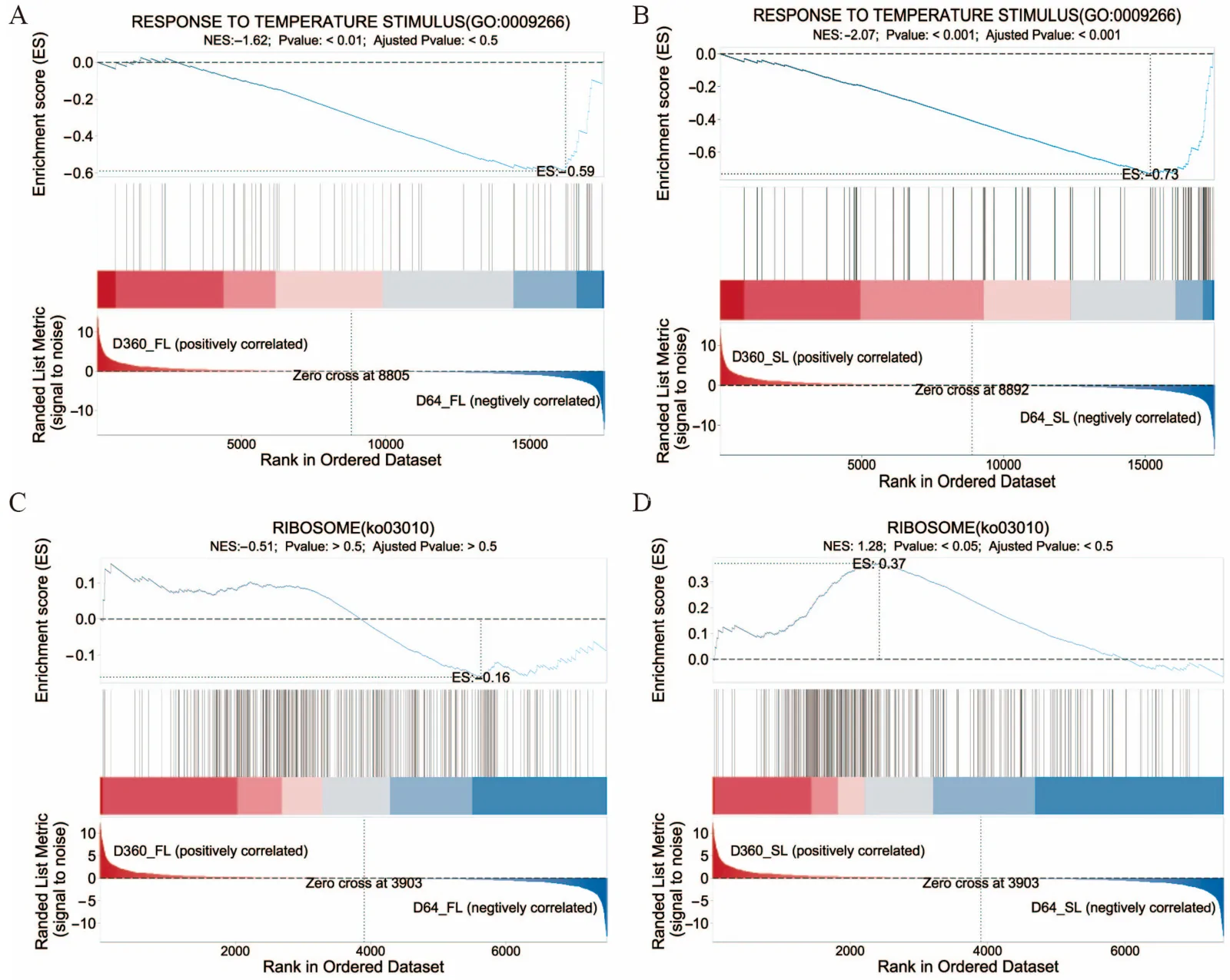
Representative GSEA enrichment plots of GO and KEGG gene sets in FL and SL. (**A**) GSEA of the GO term “response to temperature stimulus” in D64_FL versus D360_FL. (**B**) GSEA of the same GO term in D64_SL versus D360_SL. (**C**) GSEA of the KEGG pathway “Ribosome” in D64_FL versus D360_FL. (**D**) GSEA of the “Ribosome” pathway in D64_SL versus D360_SL. The normalized enrichment score (NES), nominal *p* value, and adjusted *p* value are indicated in each panel. Different colors indicate the enrichment score and ranked list metric, as shown in the figure. The dashed line indicates the zero reference level.

## Data Availability

Data are contained within the article or Supplementary Materials.

## Supplementary Materials

The following supporting information can be downloaded at: https://www.mdpi.com/article/10.3390/plants15213334/s1, Table S1: GWAS *p*-values and significance threshold.

## Abbreviations

The following abbreviations are used in this manuscript:

GWAS	Genome-wide association study
RNA-seq	RNA sequencing
LD	linkage disequilibrium
DEGs	Differentially expressed genes

## Appendix A

### Appendix A.2

**Table A1:** Summary of RNA-seq sequencing quality and mapping statistics for all samples.

Sample	Clean Reads (M)	Total Mapping Rate (%)	Unique Mapping Rate (%)	Q30 (%)
D64_FL_1	42.68	96.9	94.03	96.07
D64_FL_2	48.1	96.84	93.95	96.25
D64_FL_3	45.5	96.81	93.9	96.02
D64_SL_1	37.68	96.68	93.95	95.8
D64_SL_2	47.51	96.71	93.93	96.16
D64_SL_3	47.54	96.63	93.85	96.04
D360_FL_1	41.76	96.34	93.63	96.08
D360_FL_2	45.23	96.38	93.63	96.21
D360_FL_3	38.54	96.36	93.66	96.18
D360_SL_1	41.5	96.11	93.53	96.22
D360_SL_2	40.18	96.11	93.53	96.11
D360_SL_3	45.35	96.11	93.5	96.14

**Table A2:** Primers used for candidate gene sequencing in this study.

Gene Name	Primer Name	Forward Primer (5′-3′)	Reverse Primer (5′-3′)
*LOC123447426*	426ZF-1a	AGCCAGAAGAACCAGG	TTTGACGAGGCGATAC
*LOC123449930*	930RB-1a	CTCGGTCTCACCATCATC	GCACTTCCTTGACTATCCC
	930RB-2a	TGTGGAGAAGCGGTTC	AGCCCAAGTTGTCAGG
	930RB-3a	ACAACTCACAACAGAGGAC	ACCTGCTGCTATTACCTG
	930RB-4a	GTTGTGCCCTTCAATGAC	ACTGCCCACTTCGGTATC
	930RB-5a	AACGCCTTCTGGTTTACG	GCTGTCAGTGATTCCCAC
	930RB-6a	ATTACTTGAGTGTCCACATTCG	TGCCACCAGTTTCACG
*LOC123450878*	878CR-b	GGCTCCCTTGATAAATAC	TGCTGTGACCGAGTAAG

**Table A3:** Primers used for qRT-PCR analysis in this study.

Gene Name	Primer Name	Forward Primer (5′-3′)	Reverse Primer (5′-3′)
*LOC123447426*	RT-426	ATGGACCTGTCTCTGACCTTC	GAGACGAGGCGGCACTGTAATCG
*LOC123449930*	RT-930	GAAGTTCATCCAGTATCGCAAC	CACCTGCTGCTATTACCTTATGG
*LOC123450878*	RT-878	ATGGCCAACCCCAACAACGCT	ACCTGTAGCCGCACGAGTAG
